# Methylation-induced suppression of YAP/TAZ confers sensitivity to HDAC inhibitors in high-grade IDH mutant gliomas

**DOI:** 10.1172/jci.insight.195385

**Published:** 2025-10-09

**Authors:** Thomas K. Sears, Matthew McCord, Wenxia Wang, Alicia Steffens, Kathleen McCortney, Rahul Chaliparambil, Jann N. Sarkaria, Craig M. Horbinski

**Affiliations:** 1 Department of Neurological Surgery, Feinberg School of Medicine, Northwestern University, Chicago, Illinois, USA.; 2 Department of Pathology, University of Virginia School of Medicine, Charlottesville, Virginia, USA.; 3 Department of Radiation Oncology, Mayo Clinic, Rochester, Minnesota, USA.; 4 Department of Laboratory Medicine and Pathology, Mayo Clinic Florida, Jacksonville, Florida, USA.

**Keywords:** Cell biology, Oncology, Biomarkers, Brain cancer, Molecular pathology

## Abstract

IDH1/2 mutations (IDH^mut^) increase methylation of DNA and histones in gliomas. IDH^mut^ inhibitors are effective against low-grade IDH^mut^ gliomas, but new strategies against high-grade IDH^mut^ gliomas are needed. Although histone deacetylase inhibitors (HDACi) are ineffective against IDH^wt^ glioblastoma (GBM), their potential in IDH^mut^ gliomas has not been extensively studied. We previously established that IDH^mut^ gliomas are more sensitive to HDACi than IDH^wt^ GBM. Here we show that IDH^mut^ is associated with greater sensitivity to HDACi only in glioma, not in IDH^mut^ chondrosarcoma or cholangiocarcinoma. While HDACi induced more histone acetylation and gene regulation in IDH^mut^ glioma than in IDH^wt^ GBM, such acetylation was mostly within gene deserts, whereas IDH^mut^ glioma promoters paradoxically lost histone acetylation. Two mediators of HDACi resistance, YAP and TAZ, were methylated and suppressed in IDH^mut^ gliomas but not in other IDH^mut^ cancers. Inducing YAP or TAZ expression in IDH^mut^ gliomas conferred resistance to HDACi. Finally, belinostat extended in vivo survival only in IDH^mut^ glioma models, not in IDH^mut^ GBM models. Our findings provide a mechanistic rationale for further studies of HDACi in patients with IDH^mut^ glioma, as well as the potential use of YAP/TAZ as a biomarker of HDACi sensitivity in cancers.

## Abstract 2

Because IDH mutant gliomas suppress two important genes, YAP and TAZ, they are more sensitive to a class of drugs called HDAC inhibitors.

## Introduction

Adult-type diffuse gliomas are the most common neoplasm arising within the adult brain, affecting nearly 20,000 patients annually in the United States (1, 2). Nearly a third of these gliomas contain change-of-function point mutations in genes encoding either *isocitrate dehydrogenase* *1* (*IDH1*) or *IDH2* (together identified as IDH^mut^). IDH^mut^ gliomas are further subdivided into CNS World Health Organization (WHO) grades 2–4 astrocytomas and 2–3 oligodendrogliomas (2), all of which are less aggressive than grade 4 IDH WT (IDH^wt^) glioblastomas (GBM). Since the majority of IDH^mut^ gliomas have methylation-induced suppression of the gene encoding the DNA repair enzyme O-6-Methylguanine-DNA Methyltransferase (MGMT), the DNA alkylating agent temozolomide (TMZ) is a mainstay of therapy in these tumors (3–5). However, IDH^mut^ gliomas usually recur even after neurosurgical resection followed by adjuvant TMZ and radiation.

Whereas WT IDH1 and IDH2 oxidize isocitrate into α-ketoglutarate, IDH^mut^ reduces α-ketoglutarate into D-2-hydroxyglutarate (D2HG) (6). One prominent effect of D2HG is the inhibition of dioxygenases that normally use α-ketoglutarate as a cosubstrate. This includes dioxygenases that demethylate histones and DNA. As a result, IDH^mut^ gradually shifts the cell toward histone and DNA hypermethylation (7). Such reshaping of the epigenomic landscape appears to suppress cellular differentiation, in theory facilitating gliomagenesis (8). To combat this, small molecule IDH^mut^ enzyme inhibitors have been developed. Thus far, however, their efficacy is mostly limited to grade 2 gliomas lacking contrast enhancement on radiologic imaging (9). Furthermore, the majority of IDH^mut^ gliomas present as grades 3–4 at the time of clinical presentation — for example, at Northwestern Memorial Hospital (Chicago, Illinois), approximately 38% of newly diagnosed IDH^mut^ gliomas are CNS WHO grade 2, 49% are grade 3, and 13% are grade 4. Moreover, the tumors initially diagnosed as grade 2 invariably progress to those higher grades, even with IDH^mut^ inhibition. New strategies against high-grade IDH^mut^ gliomas are therefore urgently needed.

One approach for high-grade IDH^mut^ gliomas is to capitalize on IDH^mut^-induced epigenetic dysregulation by using drugs that target those epigenetic modifications, including modifications involving histones. Histones are protein complexes around which DNA is wrapped. When histone lysine residues within a given section of DNA are acetylated, the histones spread apart, allowing transcription factors to access the DNA. Conversely, when histone lysine residues are methylated, the histones compact together, closing off the DNA (10). Histone deacetylase inhibitors (HDACi) block the function of HDAC enzymes, thereby increasing overall histone acetylation. Since a particular histone lysine can only be either methylated or acetylated, not both, and since IDH^mut^ shifts histone lysine residues toward methylation, it follows that HDACi may counteract the epigenomic effects of IDH^mut^ on histone modifications by encouraging acetylation. Consistent with this, we previously showed that IDH^mut^ gliomas are more sensitive to HDACi in vitro, including a greater increase in global histone acetylation, compared with IDH^wt^ GBM (11). Here we extend those findings by studying the effects of HDACi on the IDH^mut^ epigenomic landscape, by exploring the molecular mediators of HDACi sensitivity, and by further evaluating the therapeutic potential of HDACi in vivo.

## Results

### IDH^mut^ is associated with sensitivity to HDACi in glioma but not in other IDH^mut^ solid tumors.

**Figure 1:**
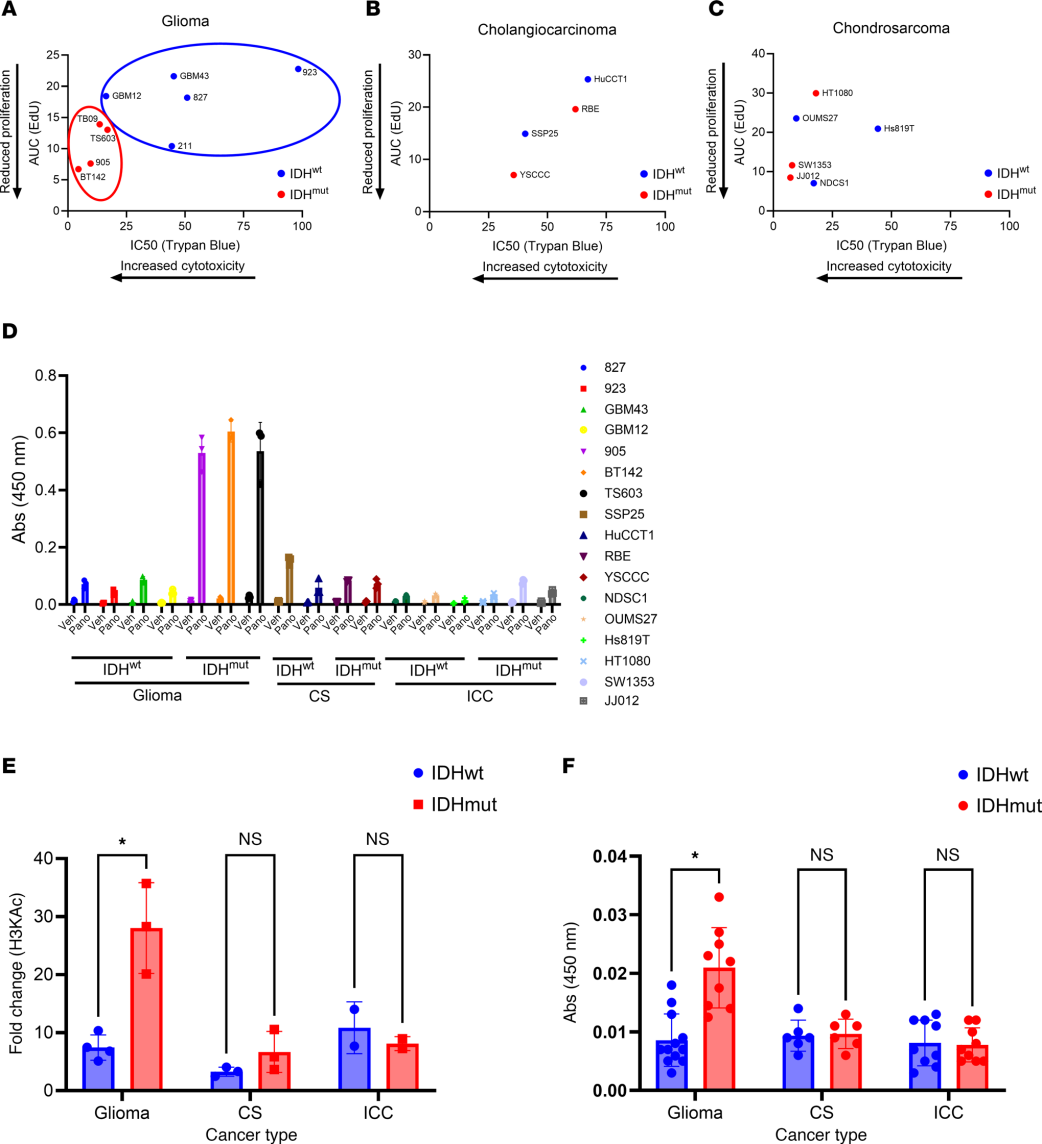
IDH^mut^ is associated with sensitivity to the HDACi panobinostat only in glioma, but not intrahepatic cholangiocarcinoma or chondrosarcoma. (**A**–**C**) Panobinostat dose-response experiments were performed via EdU proliferation and trypan blue cytotoxicity assays, and outputs were integrated onto a single plot for IDH^wt/mut^ glioma (**A**), intrahepatic cholangiocarcinoma (**B**), or chondrosarcoma (**C**). (**D**–**F**) H3KAc ELISAs were performed on a variety of IDH^wt^ and IDH^mut^ glioma, intrahepatic cholangiocarcinoma, and chondrosarcoma cell cultures treated with 10 nM of panobinostat for 24 hours. Individual cell lines are shown in **D**, while data are arranged by IDH status and cancer type in **E** showing fold changes in panobinostat-mediated H3KAc and in **F** showing baseline H3KAc. Multiple unpaired 2-tailed *t* tests with Benjamini-Hochberg correction for multiple comparisons. *P < 0.05. Data are shown as mean ± SEM (*n* = 3 biological replicates).

Consistent with our previously published data (11), IDH^mut^ is associated with a greater antiproliferative and cytotoxic response to the HDACi panobinostat in patient-derived glioma cell sources (Figure 1A). However, this was not observed in patient-derived cells representing other cancers that often contain IDH^mut^: intrahepatic cholangiocarcinoma (ICC) (Figure 1B), chondrosarcoma (CS) (Figure 1C), and acute myeloid leukemia (AML) (Supplemental Figure 2A; supplemental material available online with this article; https://doi.org/10.1172/jci.insight.195385DS1), suggesting that, aside from gliomas, IDH^mut^ is not sufficient to confer HDACi sensitivity across cancer types. Consistent with this lack of difference to HDACi sensitivity in ICC and CS, analysis of H3KAc showed that only IDH^mut^ glioma exhibits a significant difference in HDACi-mediated histone acetylation compared with its IDH^wt^ counterparts, not ICC and CS (Figure 1, D and E). Although a possible explanation involves differences in drug uptake among cell types, HPLC with tandem mass spectrometry (HPLC-MS/MS) analysis of panobinostat-treated cultures showed that IDH^mut^ gliomas actually have reduced panobinostat uptake compared with IDH^wt^ GBM (Supplemental Figure 1, F and G). Interestingly, when comparing baseline H3KAc levels between cancer types and stratifying by IDH status, only glioma showed a significant difference in H3KAc, thus suggesting a unique effect of IDH^mut^ in gliomas versus other cancers with different epigenomic landscapes (Figure 1F).

Next, we sought to determine whether this greater increase in HDACi-mediated histone acetylation within IDH^mut^ glioma cells might be mediated by increased HDAC gene expression. However, transcriptomic analyses in TCGA cohorts (grade 2/3 versus grade 4) and among our in-house glioma cultures showed that only 1 HDAC gene is consistently higher in IDH^mut^ gliomas, *HDAC5* (Supplemental Figure 3). Because the HDAC5 enzyme is a class IIa HDAC that has very little influence on histone acetylation (12), elevated HDAC5 is unlikely to account for the enhanced HDACi-induced H3KAc observed in our IDH^mut^ glioma cells.

### HDACi elicits a greater effect on gene regulation in IDH^mut^ glioma.

**Figure 2:**
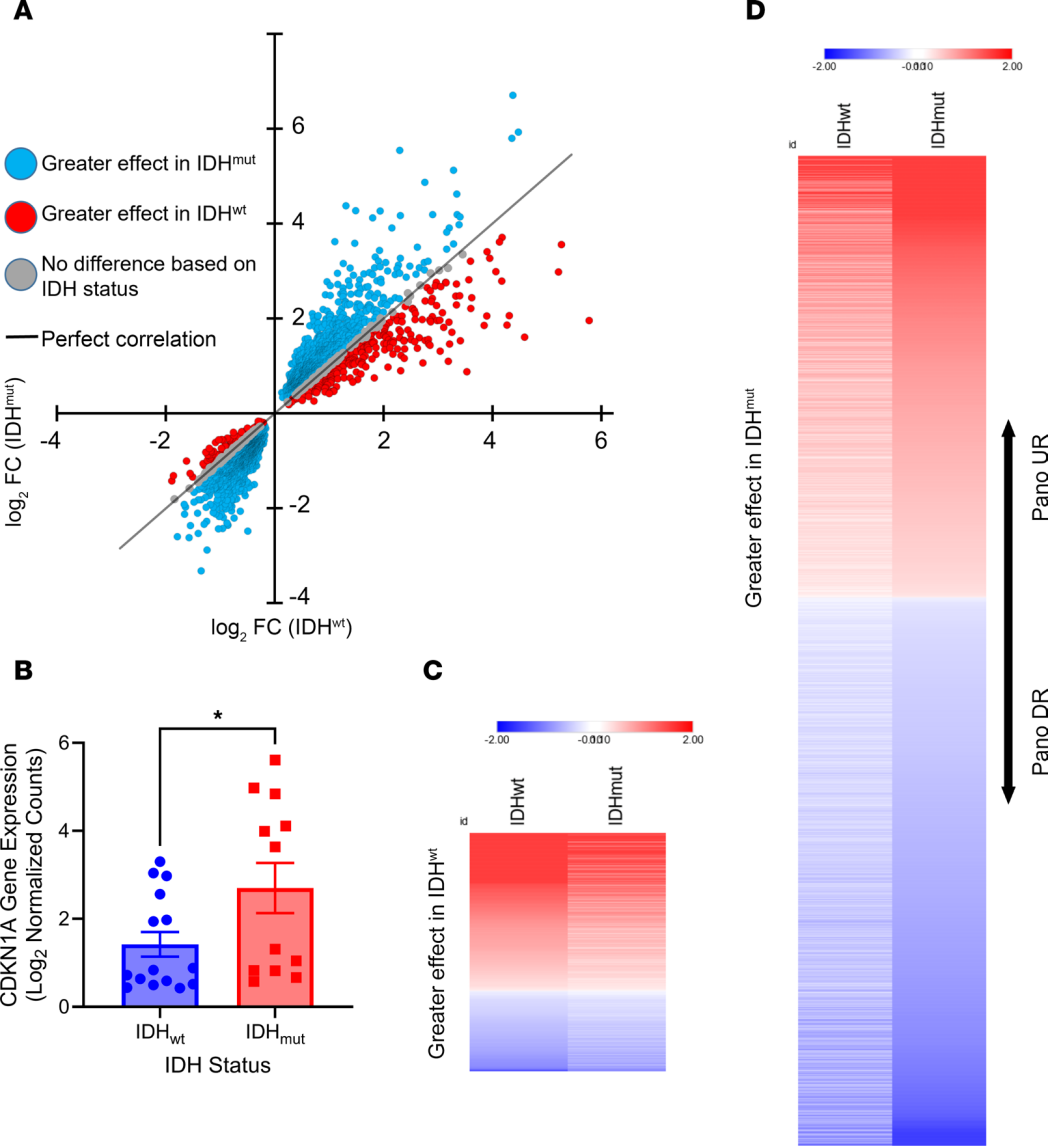
HDACi elicits a greater effect on gene regulation in IDH^mut^ glioma. (**A**) IDH^wt^ and IDH^mut^ glioma cultures were treated with 10 nM panobinostat for 24 hours before performing RNA-Seq. Differential gene expression analysis was conducted based on IDH status, and the Log_2_FC for each gene was plotted on a scatterplot to visualize panobinostat-mediated differences in gene regulation based on IDH status. (**B**) Example of one such gene, *CDKN1A*, where HDACi-mediated upregulation is greater in IDH^mut^ gliomas. Unpaired *t* test, 2-tailed. *P < 0.05. Data are shown as mean ± SEM (*n* = 3 biological replicates). (**C**) Heatmap of genes from **A** that show a greater effect in IDH^wt^ gliomas when treated with HDACi. (**D**) Heatmap of genes from **A** that show a greater effect in IDH^mut^ gliomas when treated with HDACi.

To better understand why IDH^mut^ gliomas are more sensitive to HDACi, we treated patient-derived IDH^wt^ (0827, 0923, 0211, GBM12, and GBM43) and IDH^mut^ (0905, BT142, TS603, and TB09) glioma cultures with 10 nM panobinostat (or vehicle) for 24 hours followed by RNA-Seq. While the absolute number of up- and downregulated genes do not differ based on IDH status, the magnitude of effect at each HDACi-modulated gene is much greater in IDH^mut^ glioma cultures (Figure 2A). One such gene is *CDKN1A*, which encodes the cell cycle inhibitor p21 and which, on average, exhibits ~2-fold greater upregulation in HDACi-treated IDH^mut^ glioma cultures (Figure 2B). However, this effect seems to be somewhat heterogeneous, thus precluding use as a pharmacodynamic/response biomarker. Furthermore, although the magnitude of both up- and downregulation is greater in IDH^mut^ gliomas, gene downregulation is generally more pronounced than upregulation (Figure 2, A, C, and D). The top 50 genes with greater up- or downregulation in IDH^mut^ gliomas in response to panobinostat is reported in Supplemental Table 2. This suggests that, even though HDACi are generally assumed to cause gene upregulation via histone acetylation (13, 14), IDH^mut^ glioma cells surprisingly respond to HDACi with a net downregulation of expression.

### IDH mutations influence HDACi-mediated histone acetylation at specific gene loci.

**Figure 3:**
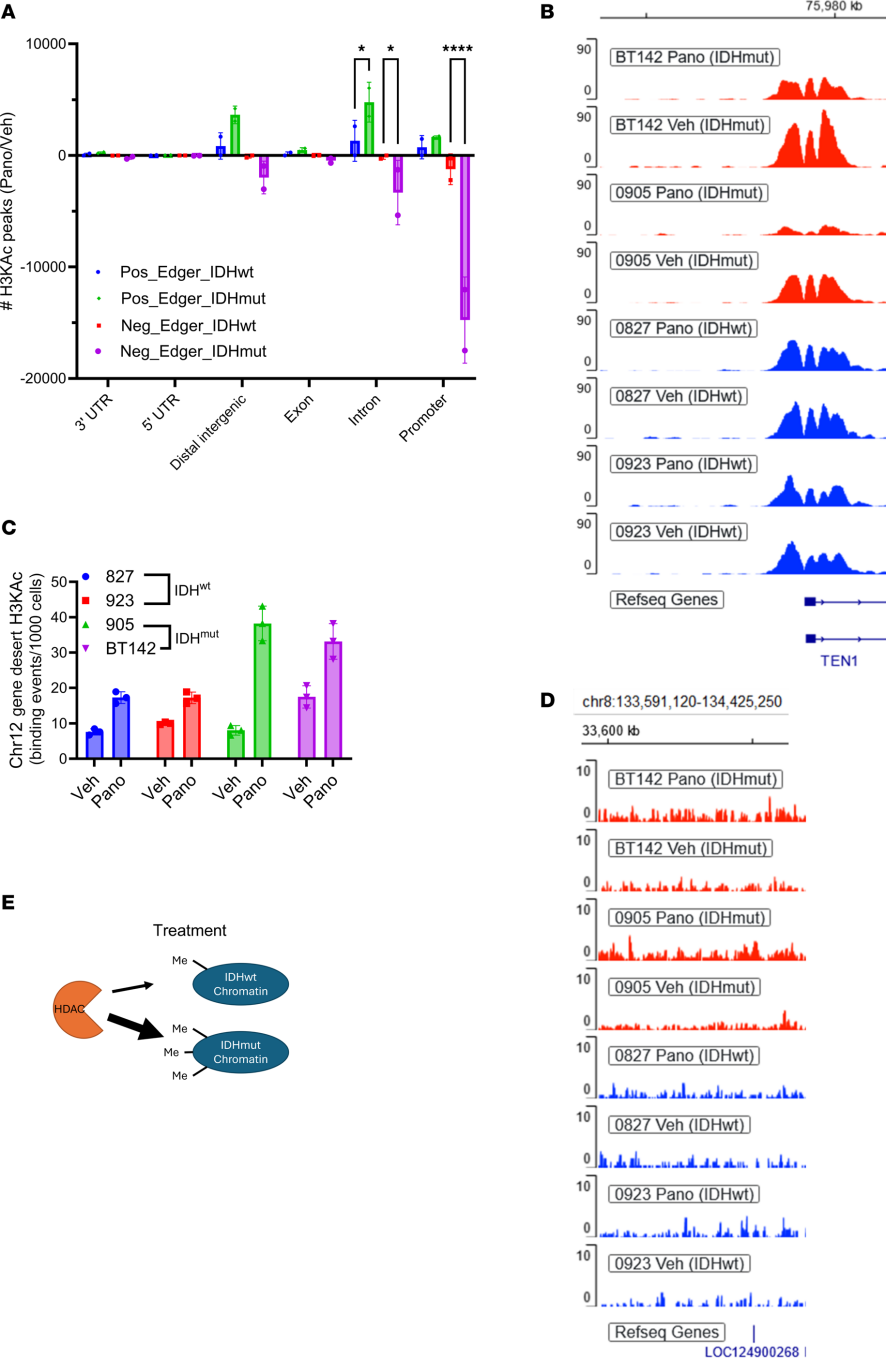
IDH mutations influence HDACi-mediated histone acetylation at specific genomic regions. (**A**) H3KAc ChIP-Seq was performed on IDH^wt^ and IDH^mut^ glioma cultures after 24 hours of panobinostat treatment. Detected H3KAc peaks were classified by genomic region and plotted as either gains or losses in H3KAc peaks based on IDH status. Two-way ANOVA with multiple comparisons. *P < 0.05, ****P < 0.0001. Data are shown as mean ± SEM (*n* = 3 biological replicates). (**B**) ChIP tracks showing a greater reduction in promoter H3KAc at the gene *TEN1* in IDH^mut^ glioma cultures. (**C**) ChIP-qPCR of panobinostat-treated IDH^wt^ and IDH^mut^ glioma cultures showing HDACi-mediated changes in H3KAc at a Chromosome 12 gene desert region based on IDH status. Data are shown as mean ± SEM (*n* = 3 biological replicates). (**D**) ChIP tracks showing a greater increase in widespread H3KAc at a Chromosome 9 gene desert region in IDH^mut^ glioma cultures treated with HDACi. (**E**) Proposed model to explain why HDACi may have a greater effect on bulk histone acetylation in IDH^mut^ gliomas. This does not exclude other possible differences, such as those involving acetyl-CoA metabolism or histone modification dynamics.

Next, we employed ChIP-Seq to analyze H3KAc throughout the genome of panobinostat- or vehicle-treated IDH^wt^ versus IDH^mut^ glioma cultures. Consistent with the paradoxical observation that HDACi-mediated gene downregulation is more pronounced in IDH^mut^ glioma, HDACi surprisingly causes more losses of H3KAc peaks at intronic and promoter regions as well as gains of intronic H3KAc peaks, in IDH^mut^ glioma cells relative to IDH^wt^ GBM cells (Figure 3A). Even though HDACi causes a net increase of global H3KAc within IDH^mut^ gliomas, it actually reduces H3KAc at promoter regions (Figure 3A). One such example is *TEN1*, which encodes a component of the CST complex involved in telomere maintenance and replication stress (Figure 3B). Juxtaposed with the RNA-Seq data (Figure 2), this suggests that the IDH^mut^ glioma epigenome responds to HDACi in a unique manner within most promoter regions, resulting in a net downregulation of gene transcription rather than upregulation.

Since HDACi still triggers more global H3KAc in IDH^mut^ gliomas versus IDH^wt^ gliomas, it suggests that this increase must be happening in nonpromoter regions, perhaps within relatively large intergenic, or “gene desert” regions. Consistent with this, ChIP–quantitative PCR (ChIP-qPCR) and ChIP tracks on a gene desert region within chromosome 12 show more H3KAc (Figure 3C and Supplemental Figure 4). Similarly, H3KAc ChIP tracks from a 20 Mb region of chromosome 8 show widespread, low-level increases in histone acetylation, primarily in the IDH^mut^ context that are not identified as H3KAc “peaks” (Figure 3D). Thus, HDACi has markedly divergent different effects on histone lysine residues within the promoter versus nonpromoter regions of IDH^mut^ gliomas. Since HDAC enzymes work as complexes that contain proteins with methyl-binding domains (15–17), it is possible that IDH^mut^-mediated genome-wide DNA and histone hypermethylation may allow broader HDAC access (Figure 3E) and that this alters their response to HDACi.

### YAP/TAZ association with HDACi sensitivity.

**Figure 4:**
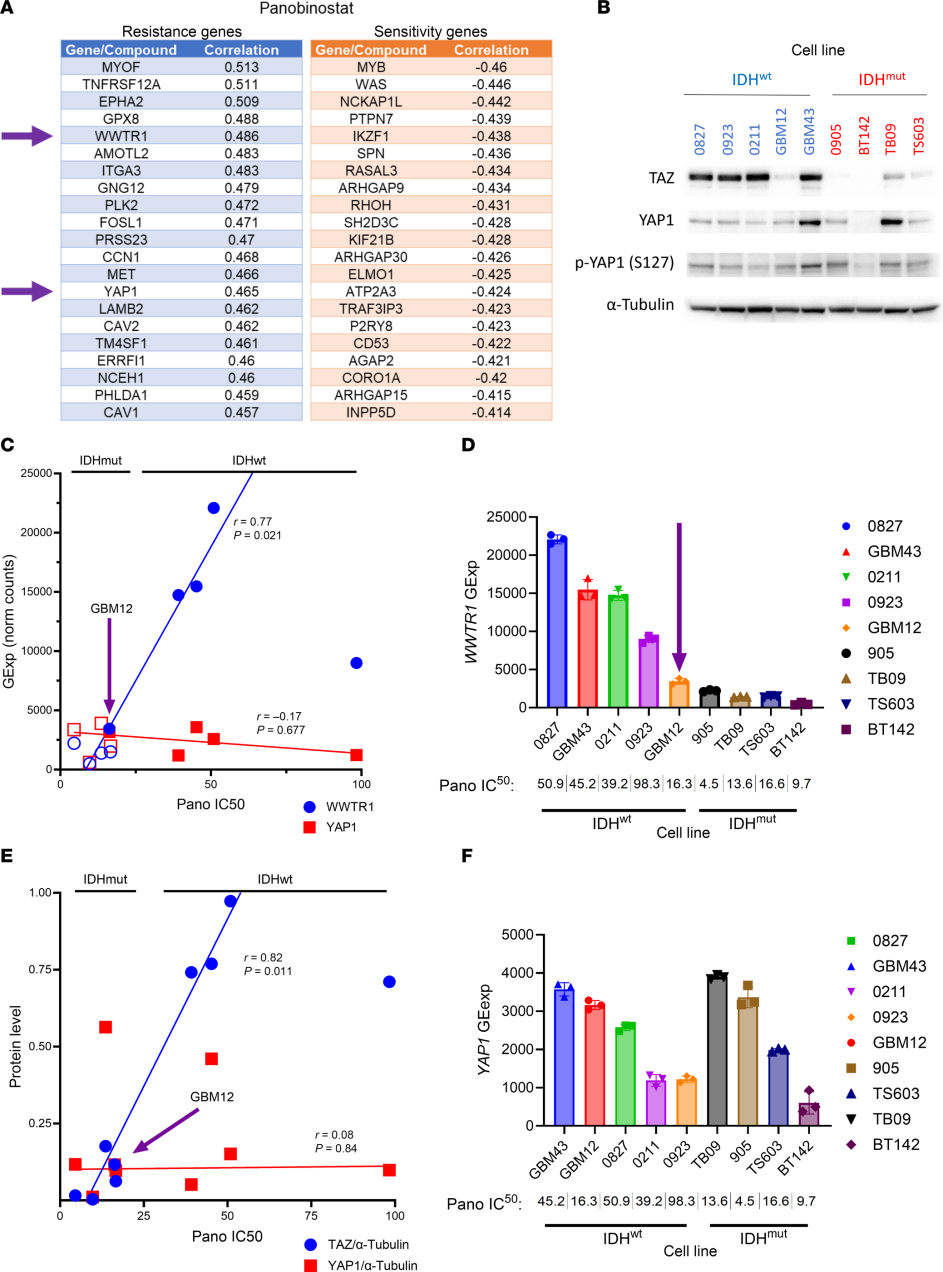
WWTR1 gene expression and TAZ protein levels are associated with HDACi sensitivity. (**A**) DepMap analysis integrating panobinostat response in cell cultures to transcriptomic gene expression data identified numerous genes associated with panobinostat resistance and sensitivity. (**B**) Western blot of IDH^wt^ and IDH^mut^ glioma cultures showing TAZ, YAP, and p-YAP (S127) protein levels. (**C**) Scatterplot evaluating correlation between *WWTR1* and *YAP1* gene expression and panobinostat IC_50_ based on IDH status. Robust linear regression with 2-tailed Spearman’s correlation analysis. Each data point represents the mean of 3 biological replicates. (**D**) *WWTR1* and *YAP1* gene expression (from RNA-Seq) and panobinostat IC_50_ for individual cell cultures with IDH status annotation. Data are shown as mean ± SEM (*n* = 3 biological replicates). (**E**) Scatterplot evaluating correlation between TAZ and YAP protein levels and panobinostat IC50 based on IDH status. Robust linear regression with 2-tailed Spearman’s correlation analysis. Each data point represents the mean of 3 biological replicates. (**F**) TAZ and YAP protein levels (from Western blot in **B**) and panobinostat IC_50_ for individual cell cultures with IDH status annotation. Data are shown as mean ± SEM (*n* = 3 biological replicates).

DepMap is a cancer dependency map that integrates multidimensional cell culture data from a variety of different tumor types (18). One application of this online resource is to screen for genes that correlate with drug sensitivity, including genes associated with panobinostat response (Figure 4A). Cross-referencing this list with our in-house RNA-Seq dataset shows several HDACi resistance genes that are expressed at higher baseline levels in our IDH^wt^ glioma cells, including *EMP1*, *WWTR1*, *EGFR*, and *AGRN* (Supplemental Figure 6A). *WWTR1*, encoding transcriptional coactivator with PDZ-binding motif (TAZ), was selected for further investigation due to its strong association with IDH status in both our RNA-Seq (Supplemental Figure 6A) and ChIP-Seq (Supplemental Figure 6B) datasets. Also included is *YAP1*, encoding Yes-associated protein 1 (YAP). YAP has substantial homology and functional redundancy with TAZ, and both YAP and TAZ are part of the Hippo signaling pathway (19). Two separate DepMap drug screens found that *WWTR1* expression inversely correlates with response to several HDACi, including panobinostat, belinostat, and vorinostat (Supplemental Table 3). We validated this at the protein level by showing that baseline TAZ, but not YAP, is elevated in most IDH^wt^ GBM cells (Figure 4B).

Next, we correlated *WWTR1* gene expression and protein levels with panobinostat IC_50_ values (Figure 4, C–E). We found a positive correlation between *WWTR1* gene expression and panobinostat IC_50_ (Figure 4, C and D), as well as between TAZ and panobinostat IC_50_ (Figure 4E). Of note, GBM12 is unusual among IDH^wt^ GBM cells in that it is sensitive to panobinostat (Figure 1A). GBM12 is also unique among IDH^wt^ cells in that its baseline expression of *WWTR1*/TAZ is relatively low (Figure 4, B–E). In contrast, neither baseline *YAP1* mRNA nor YAP protein levels correlate with panobinostat IC_50_ (Figure 4, C, E, and F), though this was obfuscated by the fact that only 2 of 9 of our glioma cultures had appreciable YAP1 protein levels.

### YAP/TAZ suppression in IDH mutant gliomas mediate HDACi sensitivity.

Since DepMap and in-house data suggest a link between *WWTR1*/TAZ and HDACi sensitivity, we determined whether adult-type diffuse gliomas contain differences in YAP/TAZ based on IDH status. Indeed, analysis of the TCGA-GBMLGG dataset showed that both IDH^mut^-codeleted (oligodendrogliomas) and IDH^mut^-noncodeleted (astrocytomas) have higher *WWTR1* and *YAP1* DNA methylation (Supplemental Figure 6, C and D), and lower *WWTR1* and *YAP1* gene expression, compared with IDH^wt^ GBM (Supplemental Figure 7, A and B). This is likely due to D2HG-mediated epigenetic repression. Consistent with the TCGA data, IHC analyses of patient-derived IDH^wt^ and IDH^mut^ gliomas showed that YAP and TAZ are lower in IDH^mut^ astrocytomas and oligodendrogliomas than in IDH^wt^ GBM (Supplemental Figure 7, C–E). In contrast, publicly available gene expression datasets for CS (Supplemental Figure 8, A and B), ICC (Supplemental Figure 8, C and D), and AML (Supplemental Figure 2, B and C) show no substantial difference in *WWTR1* or *YAP1* mRNA according to IDH status, matching their lack of differential HDACi sensitivity (Figure 1, B and C, and Supplemental Figure 2A).

**Figure 5:**
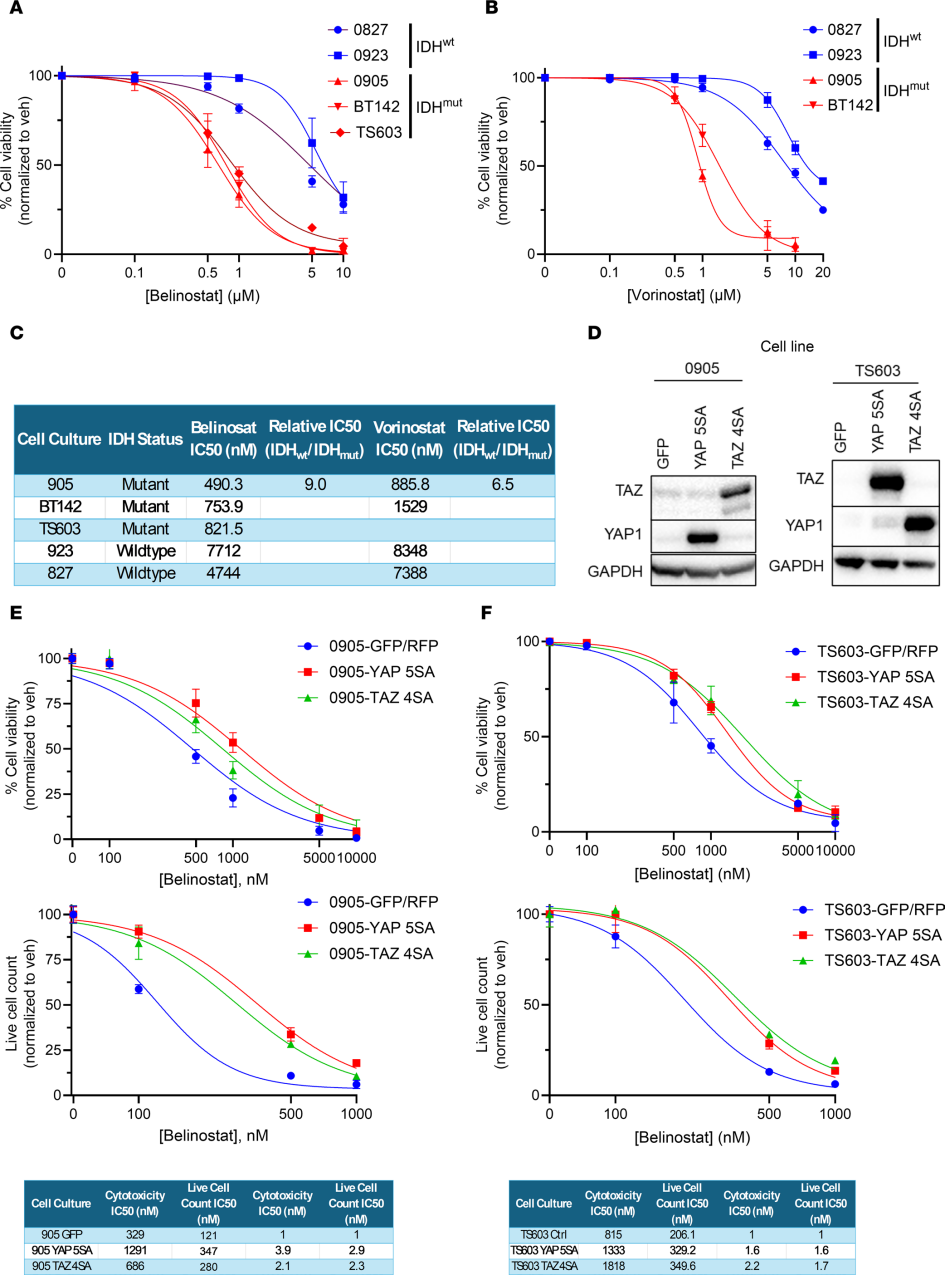
YAP/TAZ are downregulated in IDH^mut^ gliomas and affect HDACi response. (**A** and **B**) Trypan blue cell viability dose response assays in IDH^wt^ and IDH^mut^ glioma cultures treated with the HDACi belinostat (**A**) or vorinostat (**B**). (**C**) Table of belinostat (**A**) and vorinostat (**B**) IC_50_ values. (**D**) Western blot of IDH^mut^ glioma cultures with TAZ and YAP levels. (**E** and **F**) Trypan blue live cell count and cytotoxicity assays using our TAZ 4SA– and YAP 5SA–expressing 905 (**E**) and TS603 (**F**) IDH^mut^ glioma cultures treated with belinostat. IC_50_ values for live cell counts and cytotoxicity are tabulated.

Through a DepMap analysis, we identified other HDACi in which *WWTR1* gene expression correlates with sensitivity (Supplemental Table 3). In addition to panobinostat, 2 other FDA-approved hydroxamate-based HDACi were present in both DepMap drug screens: belinostat and vorinostat. Both drugs elicit a more cytotoxic response in the IDH^mut^ glioma cultures tested compared with IDH^wt^ GBM (Figure 5, A–C). Based on IC_50_ values, belinostat is twice as potent as vorinostat (Figure 5C).

While YAP and TAZ levels are lower in IDH^mut^ gliomas, and YAP and TAZ exhibit overlapping transcriptional activity, baseline TAZ appears to be more associated with HDACi response than baseline YAP in vitro, though this could be explained by the generally low YAP levels in all of our culture. Nevertheless, we therefore examined the independent roles of YAP and TAZ on HDACi response in IDH^mut^ gliomas. To do this, constitutively active TAZ (TAZ 4SA) or YAP (YAP 5SA) were expressed in 2 different patient-derived IDH^mut^ glioma cultures: 0905 and TS603. After validating YAP and TAZ expression (Figure 5D), we performed a dose-response assay with belinostat, showing that constitutively active YAP or TAZ is capable of raising belinostat IC_50_ in IDH^mut^ glioma cells to a level comparable with most IDH^wt^ GBM cells (Figure 5, E and F).

**Figure 6:**
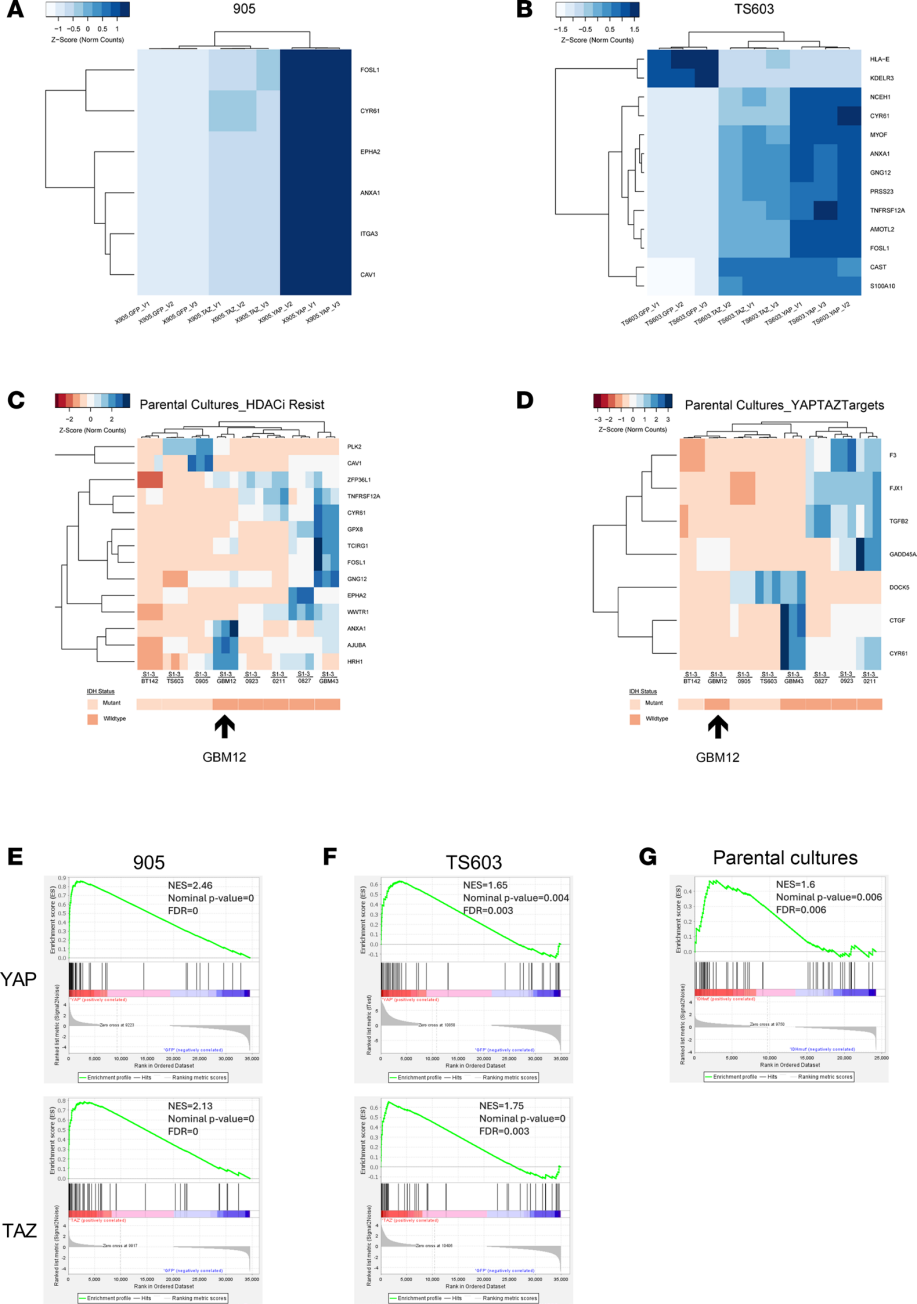
YAP or TAZ expression induces an HDACi resistance gene signature in IDH^mut^ gliomas. (**A** and **B**) Heatmap of overlapping genes identified in **A** that are associated with an HDACi resistance signature in 905 (**A**) and TS603 (**B**). (**C** and **D**) RNA-Seq was performed on our IDH^wt^ and IDH^mut^ parental cultures, genes filtered based on statistical significance and Log_2_FC > 1; then, heatmaps were produced of those gene associated with an HDACi resistance signature (**C**) or a YAP/TAZ gene target signature (**D**). (**E**–**G**) GSEA for an HDAC Resistance and an HDAC Sensitivity Gene Signature were performed on YAP 5SA– and TAZ 4SA–overexpressing 0905 (**E**), TS603 (**F**), and baseline parental cultures (**G**).

We also performed RNA-Seq on YAP- and TAZ-expressing cells to see whether YAP/TAZ affects previously described HDACi resistance and/or sensitivity gene signatures (Supplemental Table 4) (20). When assessing differentially expressed genes in TS603 and 905 IDH^mut^ glioma cells with versus without YAP or TAZ expression, and filtering genes with low expression (Mean Norm Counts <10, statistical significance *P*_adj_<0.05 and magnitude of change log_2_FC > 1 or log_2_FC < –1), numerous genes are altered in response to YAP/TAZ (Supplemental Figure 5, A and B). Some of those genes have previously been associated with either HDACi resistance or sensitivity (Supplemental Table 5) (20). For TS603 and 905 with YAP 5SA or TAZ 4SA expression, multiple genes associated with HDACi resistance are upregulated (Figure 6, A and B), whereas none of the HDACi sensitivity genes are altered based on our criteria. Of note, 905 with TAZ 4SA expression yielded substantially fewer differentially expressed genes than its YAP 5SA counterpart (Supplemental Figure 5A). This correlates with the magnitude of gene expression changes in differentially expressed HDACi resistance genes (Figure 6A), and also with the cytotoxic effects of belinostat (Figure 5E). In contrast, there are fewer differences in YAP 5SA– and TAZ 4SA–induced gene expression changes and HDACi resistance in TS603 cells (Figure 6B and Supplemental Figure 6, A and B). This may be due to more similar degrees of YAP 5SA versus TAZ 4SA expression in TS603 cells (Figure 5D).

In parental unmodified IDH^wt^ GBM cells, many known HDACi resistance genes are upregulated (Figure 6D). Cross-referencing those genes with genes that are upregulated by constitutively active YAP or TAZ expression in IDH^mut^ cells shows 3 similarities: *CYR61*, *ANXA1*, and *FOSL1* (Figure 6, B–D). Among those 3 genes, *ANXA1* and *CYR61* are even further upregulated in IDH^mut^ cells after HDACi (Table 1). Genes that are known to be downstream of YAP/TAZ are all lower in IDH^mut^ glioma cells at baseline (Figure 6E), further indicating low YAP/TAZ activity in parental IDH^mut^ glioma cells. When we tried to perform these experiments in a converse fashion using YAP1- and TAZ-KO models in our IDH^wt^ glioma cultures, we were never able to establish more than partial/hemizygous KO in both GBM43 and 0923 using 4 different TAZ gRNAs at an MOI of 30, suggesting a unique difficulty for eliminating TAZ function in IDH^wt^ glioma (not shown).

The HDACi-sensitive IDH^wt^ GBM12 cell type clustered with IDH^mut^ glioma cells when generating heatmaps via HDACi resistance genes (Figure 6C) and YAP/TAZ target genes (Figure 6D). Of note, YAP/TAZ expression does not alter the amount of bulk H3KAc in response to HDACi in either 0905 or TS603 IDH^mut^ glioma cells (Supplemental Figure 5, C and D). This aligns with H3KAc data showing that YAP/TAZ^lo^ GBM12 treated with HDACi yields a similar modest increase in H3KAc, as in other IDH^wt^ cells (Figure 1D).

As an alternative approach to investigating the role of YAP and TAZ in regulating HDAC resistance and sensitivity signatures, we employed Gene Set Enrichment Analysis (GSEA) using custom gene sets found in Supplemental Table 4. This allows for a more rigorous statistical evaluation of the gene sets and also does not require filtering of low expressed genes. Consistent with our previous analysis, both YAP and TAZ overexpression in our 0905 and TS603 IDH^mut^ glioma cultures displayed significant enrichment of the HDACi Resistance Gene Set, and this was also observed for our parental cultures (Figure 6, E–G), further highlighting the importance of YAP and TAZ in activating genes involved in HDACi resistance. Interestingly, our GSEA found that 0905 with both YAP and TAZ overexpression also induces a significant reduction in the expression of genes in the HDACi Sensitivity Gene Set, whereas this was not true for TS603, though we observed a similar trend in our parental cultures which wasn’t statistically significant (Supplemental Figure 5, E–G). This further exemplifies the diverse action that YAP and TAZ can have on HDACi response where it seems that, in certain contexts, they not only upregulate genes involved in HDACi resistance but also downregulate genes that promote HDACi sensitivity.

### IDH status predicts response to HDACi in intracranial xenograft models of glioma.

**Figure 7:**
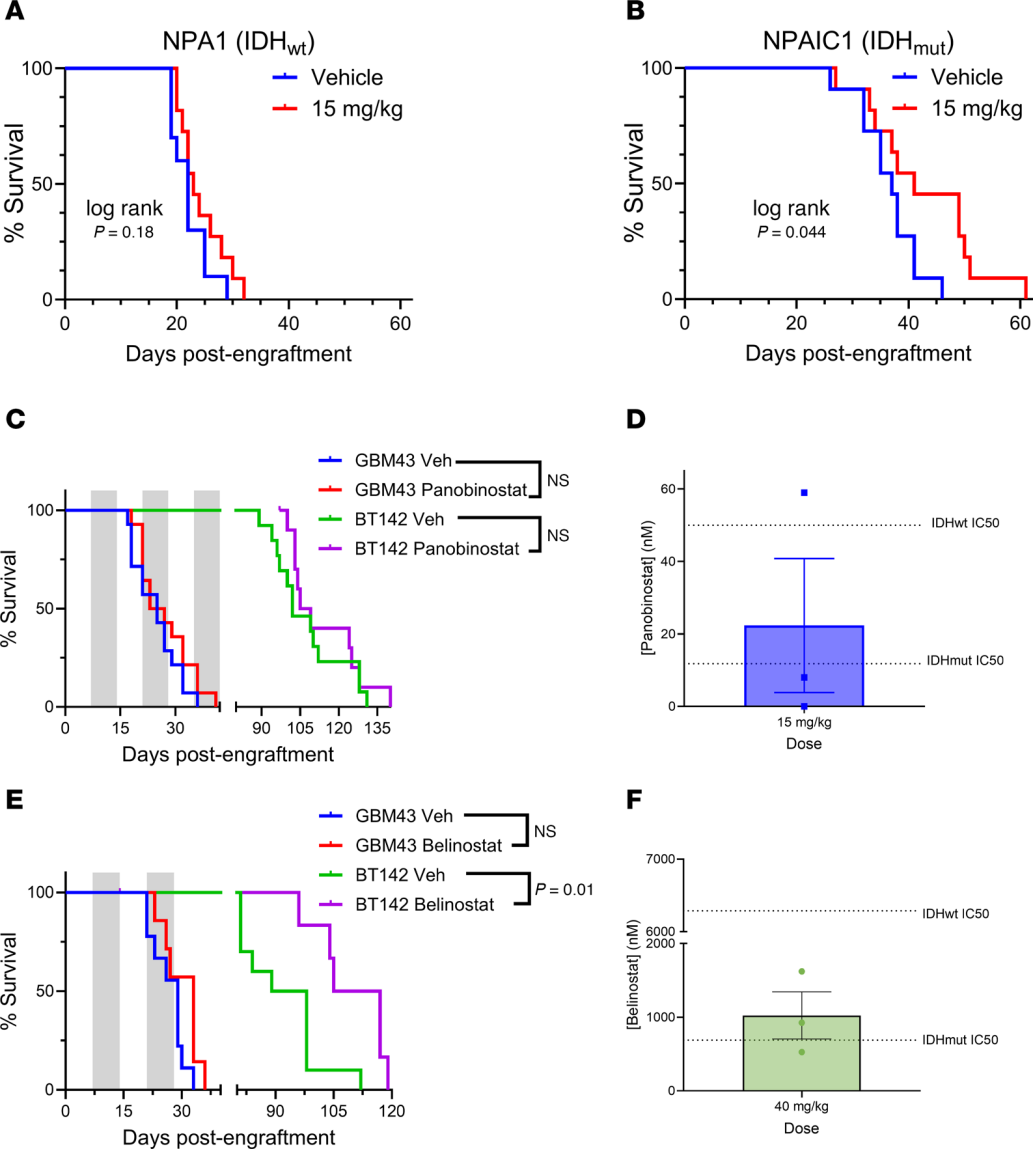
IDH^mut^ predicts response to HDACi in intracranial xenograft models of glioma. (**A** and **B**) Kaplan-Meier survival analysis of C57BL/6 mice showing the in vivo response to 15 mg/kg panobinostat i.p., 3×/week using syngeneic intracranial models of isogenic murine IDH^wt^ (**A**) or IDH^mut^ (**B**) glioma cultures. Mantel-Cox log-rank test, *n* = 12 per group. (**C**) Kaplan-Meier survival analysis of NSG mice showing the in vivo response to 15 mg/kg panobinostat i.p., 3×/week using intracranial xenograft models of patient-derived IDH^wt^ and IDH^mut^ gliomas. Mantel-Cox log-rank test, *n* = 12 per group. (**D**) Quantitative analysis of panobinostat brain uptake via tandem mass spectrometry after administration of 15 mg/kg panobinostat i.p. Data are shown as mean ± SEM (*n* = 3 biological replicates). Dotted lines represent average cell viability IC_50_ for panobinostat in our IDH^wt^ and IDH^mut^ glioma cultures. (**E**) Kaplan-Meier survival analysis of NSG mice showing the in vivo response to 40 mg/kg belinostat i.p., 5×/week BID, using intracranial xenograft models of patient-derived IDH^wt^ and IDH^mut^ gliomas. Mantel-Cox log-rank test, *n* = 12 per group. (**F**) Quantitative analysis of panobinostat brain uptake via tandem mass spectrometry after administration of 40 mg/kg belinostat i.p. Data are shown as mean ± SEM (*n* = 3 biological replicates). Dotted lines represent average cell viability IC_50_ for belinostat in our IDH^wt^ and IDH^mut^ glioma cultures.

In preclinical trials, we first investigated the therapeutic potential of panobinostat in a syngeneic orthotopic mouse model of glioma with isogenic IDH^wt^ (NPA1) or IDH^mut^ (NPAIC1) (21). In this model, *WWTR1*, but not *YAP1*, mRNA is lower in IDH^mut^ NPAIC1 cells versus IDH^wt^ NPA1 cells (Supplemental Figure 9). A maximally tolerated dose of panobinostat (15 mg/kg), produces a survival benefit only in NPAIC1 IDH^mut^ glioma, whereas NPA1 IDH^wt^ tumors show no response (Figure 7, A and B). In a patient-derived orthotopic xenograft setting, neither IDH^wt^ GBM43 nor IDH^mut^ BT142 gliomas respond to panobinostat (Figure 7C). In the setting of GBM43 glioma xenografts, systemic administration of panobinostat does not consistently achieve therapeutic levels within tumors (Figure 7D).

We therefore tested belinostat, which remains FDA approved and has superior blood-brain barrier penetrability and dosing/safety profile versus panobinostat (22, 23). Indeed, belinostat extends the median survival of mice engrafted with IDH^mut^ BT142 but not IDH^wt^ GBM43 (Figure 7E). Using the same fast-growing GBM43 intracranial xenograft model we employed for our panobinostat studies, we observed that belinostat reaches an average intratumoral concentration of 1,000 nM, exceeding its in vitro IC_50_ for IDH^mut^ glioma but still well below the IC_50_ for IDH^wt^ GBM (Figure 5C). This concentration is 50× higher than the average intratumoral concentration of panobinostat (Figure 7D), even though the in vivo dose of belinostat is only 2.7× higher. This suggests that belinostat tumor uptake is greater than panobinostat and further exemplifies the superior translational potential of belinostat over panobinostat for the treatment of IDH^mut^ glioma. One limitation of this approach is that, since we used GBM43 xenografts for these studies, the magnitude of tumor uptake may be slightly different than if we used BT142. Nevertheless, the relative amount of drug penetration would likely be similar, and the drug uptake data for GBM43 (Figure 7, D and F) correlates well with our efficacy data for BT142 (Figure 7, C and E)

## Discussion

Although IDH^mut^ gliomas are comparatively less aggressive than their IDH^wt^ GBM counterparts, patients still only have a median survival of ~34 months (24). Therefore, new treatment options are needed for these patients. This includes revisiting therapies that failed in older trials against IDH^wt^ GBMs, provided that a mechanistic rationale exists. Our data show that IDH^mut^ gliomas are more sensitive to hydroxamate-based HDACi, including panobinostat, vorinostat, and belinostat. This effect is associated with YAP/TAZ, a primary effector of the Hippo signaling pathway, and also with a proportionately greater increase in HDACi-mediated H3KAc. In contrast, other IDH^mut^ cancers are not more sensitive to HDACi, do not experience more upregulation of HDACi-induced H3KAc, and show no difference in baseline YAP or TAZ expression relative to their IDH^wt^ counterparts. This aligns with our previous work showing that, while IDH^mut^ causes genomic hypermethylation, the exact kinds of genes that are hypermethylated varies substantially according to tumor type (25). Thus, results of epigenomic modulators in one type of IDH^mut^ cancer do not automatically apply to other IDH^mut^ cancers (24, 26–28). This supports the idea that, other than in glioma, IDH^mut^ is not sufficient to confer sensitivity to HDACi across cancer types.

Within the Hippo signaling pathway, YAP and TAZ are coactivators in collaboration with TEA domain (TEAD) transcription factors (29, 30). When the Hippo pathway is activated, YAP/TAZ are sequestered or degraded, and cells stop growing. Thus, the Hippo pathway prevents tissues and organs from becoming too large (31). YAP/TAZ are increased in many cancers, thus enhancing self-renewal and proliferation (30, 31). While YAP and TAZ are paralogs of each other, and have many overlapping activities, they bind TEADs independently of each other and have some distinct functions. For example, YAP tends to have broader effects on cell size, metabolism, and proliferation than TAZ (32). In our IDH^wt^ and IDH^mut^ cell models, baseline TAZ correlates better with HDACi sensitivity than does YAP. However, either constitutively active YAP or TAZ confers resistance to HDACi in IDH^mut^ glioma cells, on par with what is observed in IDH^wt^ GBM cells. Constitutively active YAP and TAZ in our IDH^mut^ glioma cultures also upregulate similar HDACi resistance genes as are expressed in IDH^wt^ GBM. This suggests that the lack of correlation between HDACi resistance and *YAP1* expression in our parental cultures might be explained by the very low expression of *YAP1* in all but 2 of 9 of our glioma cultures. Thus, while TAZ may be more important for HDACi resistance in many cancers, YAP may also confer resistance when sufficiently upregulated.

Through a pharmaco-transcriptomic analysis of publicly available drug screening data for HDACi using 659 cell lines, others identified 46 genes correlating with HDACi sensitivity and 53 genes correlating with HDACi resistance (20). From an in silico analysis of those HDACi sensitivity and resistance genes, the authors predicted (but did not prove) that, out of all TCGA datasets, IDH^mut^ gliomas should be the most sensitive to HDACi. Conversely, they also found that YAP-driven non-small cell lung cancers have a high HDACi resistance signature and are resistant to HDACi. In our study, GBM12 was an outlier among IDH^wt^ GBMs in being sensitive to HDACi. Perhaps not coincidentally, this cell source also expresses less baseline YAP and TAZ relative to other IDH^wt^ GBM. Inducing constitutively active YAP and TAZ made IDH^mut^ glioma cells just as resistant to HDACi as IDH^wt^ GBM. The specific downstream effectors of YAP/TAZ-mediated resistance to HDACi are the subject of ongoing investigation, but they may include *CYR61* and *ANXA1*, among others. These data not only indicate that methylation-induced suppression of YAP and TAZ render IDH^mut^ glioma cells more vulnerable to HDACi, but that YAP/TAZ might serve as a predictive biomarker for HDACi response across all cancers.

Although YAP/TAZ conferred HDACi resistance in our IDH^mut^ cells, they did not suppress overall HDACi-mediated histone acetylation. This suggests that the increased effect of HDACi on IDH^mut^ glioma cells may be due more to YAP/TAZ suppression than to global changes in histone acetylation marks. Furthermore, although HDACi does trigger the expected increase in global histone acetylation in IDH^mut^ gliomas, this surprisingly applies only to large intergenic and gene desert regions. In contrast, smaller promoter regions within the IDH^mut^ genome tend to show reduced histone acetylation after HDACi, and subsequently reduced gene transcription. This is the opposite of what HDACi is proposed to do in most cancers (13, 14). The reasons for this are unclear, but since HDAC complexes contain proteins with methyl-binding domains (15–17), those complexes may function differently in an IDH^mut^ genome with widespread hypermethylation. It is also possible that IDH^mut^ alters baseline acetyl-CoA metabolism and/or histone modification dynamics. Future studies will assess these possibilities, including colocalization of HDAC genomic occupancy and DNA methylation in IDH^mut^ gliomas using chromatin accessibility assays. While no obvious HDAC candidate has yet emerged based on preferential baseline expression in IDH^mut^ gliomas, others have shown that IDH^mut^ gliomas are especially reliant on HDAC1 and HDAC6 activity (33). Co-occupancy analysis of HDACs and methyl-binding proteins, as well as isoform-specific knockdowns of specific HDACs, will therefore also be featured in our follow-up study.

Given the prominent roles of YAP/TAZ in facilitating IDH^wt^ GBM malignancy (34, 35), their hypermethylation and suppression in IDH^mut^ gliomas may also contribute to the reduced aggressiveness of IDH^mut^ gliomas, apart from any effects on HDACi sensitivity. Our data show that none of the other major IDH^mut^ cancers suppress YAP/TAZ, and IDH^mut^ is not a favorable prognostic marker in those other cancers (25). Although high YAP/TAZ renders most IDH^wt^ GBM cells insensitive to HDACi, others have shown that such expression makes IDH^wt^ GBM cells more sensitive to verteporfin, an inhibitor of YAP/TAZ/TEAD-mediated transcription (30, 36).

Since aberrant histone modification patterns play key roles in cancer, the strategy of HDACi has been evaluated against many cancers, including adult-type diffuse gliomas, with disappointing results thus far (37–45). Indeed, based on their generally high YAP/TAZ, IDH^wt^ GBMs are among the worst candidates for HDACi (20). However, clinical studies have either focused exclusively on IDH^wt^ GBM (37–43) or included too few IDH^mut^ gliomas to support meaningful statistical analyses of their responses to treatment (45, 46). The few patients with IDH^mut^ glioma who received HDACi treatment showed encouraging results (47). For example, a patient with an IDH^mut^ grade 4 astrocytoma who was treated with belinostat showed no further progression over 16 months and experienced neurocognitive improvement (48). A single-arm phase 2 trial of mostly IDH^wt^ GBM included 10 patients with IDH^mut^ glioma, who had 70% progression-free survival after 6 months (PFS6) on panobinostat and the VEGF-A inhibitor bevacizumab, versus less than 30% PFS6 in IDH^wt^ patients with GBM (49). But that study focused only on patients whose gliomas recurred after standard radiotherapy and TMZ, and it lacked matched control groups. In our orthotopic preclinical trials, the HDACi belinostat was active against IDH^mut^ glioma but not IDH^wt^ GBM, aligning with our in vitro data. Interestingly, belinostat tumor concentrations were ~50× higher than panobinostat even though the in vivo drug dosing was only 2.7× higher, suggesting superior drug uptake and pharmacokinetic properties for belinostat over panobinostat. Therefore, while there is no demonstrable role for HDACi in treating IDH^wt^ GBM, the same cannot be assumed for IDH^mut^ gliomas, particularly for belinostat.

Our data showing unique sensitivity of IDH^mut^ gliomas to HDACi has been confirmed by others in recent studies (33, 50–52), demonstrating a growing interest in exploiting this therapeutic vulnerability. A drug screen by one group showed that HDACi are among the most selective compounds at inhibiting IDH^mut^ glioma cell growth (33). Another group showed that HDACi and tazemetostat, an Enhancer of Zeste 2 (EZH2) inhibitor, synergize against a murine model of IDH^mut^ glioma (51). A third group suggested that IDH^mut^ may lower the amount of acetyl-CoA available to acetylate histones and that this might account for the increased HDACi activity against IDH^mut^ gliomas (52). However, those data were derived from the acute expression of exogenous IDH1 R132H in IDH^wt^ HEK and GBM cells, and they did not study patient-derived gliomas with long-term endogenous expression of IDH^mut^. Furthermore, if acetyl-CoA depletion accounted for HDACi sensitivity, one might expect increased HDACi sensitivity across all IDH^mut^ cancers, but our data suggest the epigenomic suppression of YAP/TAZ may be more important.

In conclusion, these data establish a mechanistic rationale for repurposing HDACi in patients with high-grade IDH^mut^ gliomas, perhaps as part of a combinatorial approach employing other modalities like chemoradiation. More broadly, YAP/TAZ may serve as useful predictive biomarkers for HDACi response across a wide range of cancers. As our knowledge of molecular subtypes of cancer evolves and matures, and the field strives toward personalized tumor-specific approaches, such opportunities to reconsider previously discarded treatments are likely to continue emerging.

## Methods

### Sex as a biological variable.

Patient-derived gliomas used in tissue-based studies originated in male and female patients from Northwestern University Nervous System Tumor Bank, collected under an IRB-approved protocol (no. STU00095863). All animal studies were done under an IACUC-approved protocol (no. IS00002518). Our study examined male and female animals, and similar findings are reported for both sexes.

### Cell culture models.

Glioma, cholangiocarcinoma, and CS cell culture models were acquired via the sources listed in Supplemental Table 1. Tumor subtype, grade, IDH status, and cell culture media recipes are also specified in Supplemental Table 1. Except for TB09, all gliomas were cultured in defined, serum-free glioma stem cell media (GSCM). The recipe for this GSCM is described in Supplemental Table 1. R132H IDH1 expression in these glioma cultures was validated by Western blot, and D2HG production in all cultures was quantified by a D2HG Assay Kit (catalog MAK320) (Supplemental Figure 1, A–E). Nos. 0827, 0923, 0211, and 0905 glioma cultures were provided as a gift from Kevin Woolard (UC Davis, Davis, California, USA). TB09 glioma cultures were provided as a gift by Hai Yan (Duke University, Durham, North Carolina, USA). TS603 glioma cultures were provided as a gift from Timothy Chan (Cleveland Clinic, Cleveland, Ohio, USA). JJ012 CS cultures were provided as a gift from Joel Block (Rush University, Chicago, Illinois, USA). NDCS1 CS cultures were provided as a gift from Akira Agose (Niigata University, Niigata, Japan).

### Western blotting.

Briefly, cell lysates were prepared by resuspending frozen cell pellets in RIPA buffer containing 1x EDTA-free Protease Inhibitor Cocktail (Thermo Fisher Scientific, catalogs 89901 and 87785) and then sonicated on a QSonica Q800R3 water bath sonicator (catalog Q800R3-110) for 2 cycles of 20s on/20s off sonication at 50% amplitude. Sonicated cell pellets were cleared of insoluble material via centrifugation and then soluble protein concentrations evaluated using Pierce’s Detergent Compatible Bradford Assay (catalog 23246). Proteins were separated on 4-12% SDS-PAGE gels for 1 hour at 200V, and then and transferred to 0.22 μm PVDF membranes at 30 V for 1 hour. Membranes were blocked with StartingBlock T20 Blocking Buffer (Thermo Fisher Scientific, catalog37543) and incubated with primary antibodies (1:1,000 dilution in 5% BSA dissolved in TBST) at 4°C overnight, followed by room temperature incubation with respective secondary antibodies (goat anti-rabbit, Cell Signaling Technology #7074; goat anti-mouse, Cell Signaling Technology #7076) in blocking buffer at concentration of 1:10,000 for 60 minutes. Membranes were imaged with SuperSignal West Pico PLUS Chemiluminescent reagents (Thermo Fisher Scientific #24580) using a BioRad ChemiDoc imaging system. The following primary antibodies were used in this study: anti-GAPDH (catalog 2118), anti-R132H IDH1 (catalog DIA-H09), anti-YAP1 (catalog 14074), anti-p-YAP1 (S127; catalog 13008), anti-TAZ (catalog 72804). All antibodies were purchased from Cell Signaling Technology except for the anti-R132H IDH1 antibody, which was purchased from Dianova.

### In vivo PDX engraftment and treatment.

Patient-derived GBM43 and BT142, as well as murine NPA1 (IDH^wt^) and NPAIC1 (IDH^mut^) (21), were used to test antitumor effect of HDACi in vivo. NPA1 and NPAIC1 murine glioma models were kindly provided as a gift from Dr. Maria Castro, University of Michigan, Ann Arbor, MI. Briefly, tumor spheres were cultured in defined, serum-free media. Male and female NSG mice aged 8-10 weeks received intracranial injections of 50,000 (BT142) or 100,000 (GBM43) tumor cells into right frontal lobes. Groups of engrafted mice received i.p. administration of panobinostat, belinostat, or vehicle (5% DMSO, 5% Tween 80, 40% PEG300 in water for panobinostat; 5% DMSO and 25 mg/mL L-arginine in DPBS for belinostat). Panobinostat (15 mg/kg) was administered 3x/week MWF, whereas belinostat (40 mg/kg) was administered 5x/week BID M-F. Dosing regimens were 1 week on/1 week off for both panobinostat (up to 4 cycles) and belinostat (2 cycles). Panobinostat (catalog HY-10224) and belinostat (catalog HY-10225) were purchased from MedChemExpress.

### In vitro cytotoxicity and live cell count assays.

Glioma cultures were seeded in 12-well tissue culture plates at 50,000 cells per well, using defined, serum-free GSCM. Cells were treated with drug (panobinostat, belinostat, or vorinostat) for 5 days 24 hours after seeding. Drug stocks were prepared in DMSO as 1000x concentrates. After treatment, cells were pelleted via gentle microcentrifugation, supernatant aspirated, and spheres disassociated with 0.05% trypsin. After trypsin neutralization, cells were centrifuged and resuspended to a final volume of 50 μL of media. From each tube, 10μL of suspension was mixed with an equivalent volume of trypan blue, and cell death (percent live cells) and live cell counts were quantified with a Countess II Automated Cell Counter (Invitrogen #C10283). For each condition, 3 biological replicates were assessed in this manner.

### EdU proliferation assay.

Cellular proliferation was assessed independent of cytotoxicity using early time point EdU dose-response assays. These assays were conducted via Flow Cytometry using the BD Pharmingen 647 Click Proliferation Kit (catalog 565456) following the manufacturer’s protocol. Briefly, 500,000 cells were plates into wells of a 6-well plate and treated with 0.1% DMSO vehicle, 10 nM, or 50 nM panobinostat. Untreated background controls were also included to assist in establishing forward and side scatter profiles for each cell culture. After 48 hours treatment exposure, cells were pulsed with 10 μM EdU for 2.5 hours, and then trypsinized to form single cell suspension. Cell suspensions were then fixed in 4% paraformaldehyde and frozen in freezing media (10% DMSO and 90% growth media). Cells were then stained for EdU content, strained into flow cytometry tubes, and then the red laser was used to assess EdU uptake in each sample via a BD LSRFortessa I Cell Analyzer. Cell aggregates were removed via gating of the linear portion of cells when plotted as FSC-H vs FSC-A. EdU-positive and -negative cells were differentiated by establishing gates using cultures that did not receive any EdU treatment but still received the EdU staining cocktail.

### RNA extraction and RNA-Seq.

Glioma cultures were treated with vehicle or HDACi (10 nM panobinostat; 500 nM belinostat) for 24 hours prior to sample collection. These independent biological replicates (vehicle or HDACi-treated cell cultures) were collected and RNA was extracted using an RNeasy Plus Mini Kit (catalog 73134). RNA concentration and quality was verified using a NanoDrop 2000 Spectrophotometer, and then samples were submitted for sequencing to Northwestern’s sequencing core (NUSeq). Bioinformatics support was also provided by NUSeq. Briefly, samples were sequenced via 1 × 100 single-end reads on a Complete Genomics DNBSEQ-G400 FCL and then FASTQC performed to evaluate sequencing quality. Reads were then mapped to the GRCh38 genome (TopHat2), gene transcripts quantified (HTSeq), and then differential gene expression analysis performed (deseq2). Differential gene expression data generated by deseq2 was filtered based on the following criteria to eliminate low expressed genes and identify genes with statistically significant and high-magnitude changes in expression: Average normalized counts > 10, *P*_adj_ < 0.05, and Log_2_FC > 1 or Log_2_FC < –1. For GSEA, HDACi Resistance and Sensitivity Signature were annotated, Gene Set Permutation Type, a weighted enrichment statistic, and a Signal2Noise metric for ranking genes. Datasets were unfiltered prior to GSEA.

### ChIP-Seq.

Glioma cultures were treated with vehicle or HDACi (10 nM panobinostat) for 24 hours prior to sample collection. These vehicle or HDACi-treated cell cultures were pelleted and ChIP DNA isolated using Active Motif’s ChIP-IT High Sensitivity Kit (catalog 53040). Spike-in normalization methods were utilized via use of Active Motif’s Spike-in Antibody (catalog 61686) and Chromatin (catalog 53083). Active Motif’s ChIP-IT qPCR Analysis Kit (catalog 53029) was used to validate H3KAc enrichment using positive control primers (GAPDH) and negative control primers (Chromosome 12 gene desert). Sonication was performed on our QSonica Q800R3 water bath sonicator (catalog Q800R3-110) after optimization of sonication conditions for proper chromatin fragmentation. The antibody used for our ChIP reactions was Active Motif’s pan-acetyl H3KAc antibody (catalog 39139). Samples were submitted for sequencing to NUSeq. Bioinformatics support was also provided by NUSeq. Briefly, FASTQ sequence files were evaluated for quality and trimmed via FastQC and Trim Galore!, respectively. bowtie2 was then used to align trimmed filed to the GRCH38 genome. Peaks were called using MACS2, and then differential peak analysis performed using DiffPeaks. To generate ChIP tracks, BIGWIG was used prior to visualization on the IGV web application.

### Histone acetylation ELISA.

To assess the impact of HDACi on bulk histone acetylation, Cell Signaling Technology’s PathScan H3KAc Sandwich ELISA kit was utilized (catalog 7232). Briefly, cell cultures were treated with 10 nM panobinostat for 24 hours and then flash frozen in liquid nitrogen. Vehicle- and panobinostat-treated cell pellets underwent histone extraction via Epigentek’s EpiQuik Total Histone Extraction Kit (catalog OP-0006-100), and then the protocol for CST’s H3KAc ELISA was followed to evaluate differential impacts on histone acetylation based on IDH status.

### Validation of D2HG levels in cell cultures.

Verification of IDH^mut^-mediated D2HG production was conducted using Sigma-Aldrich’s D2HG Assay Kit (MAK320). Briefly, IDH^wt^ and IDH^mut^ cell cultures were pelleted on ice and then flash frozen in liquid nitrogen prior to storage at –80°C. Samples were briefly sonicated (2 × 20s at 50% amplitude) in RIPA buffer using a QSonica Q800R3 sonicator, and then D2HG extracted and quantified following the manufacturer’s protocol. Separate, identical cell pellets from each sample were used to quantify protein concentrations using a Bradford assay (catalog 23246) and then used to normalize D2HG levels in each sample.

### Assessment of panobinostat and belinostat brain uptake via targeted UHPLC-MS/MS.

To verify uptake of the HDACi panobinostat or belinostat into the murine brain, we utilized UHPLC-MS/MS in brain tissues of mice treated with 15 mg/kg of panobinostat or 40 mg/kg for belinostat i.p. in 3 NSG mice bearing IDH^wt^ GBM43 intracranial xenografts. Five control mice were included that only received vehicle i.p. Whole brains of vehicle- and HDACi-treated mice were harvested and then micro-dissected on ice under a microscope to remove nontumor bearing brain tissue and then flash-frozen in liquid nitrogen. Forty-five mg of dissected brain tumor tissue was then homogenized with a pestle and mixed with aqueous L-arginine buffer (100 mg/kg). Samples were then placed on a thermomixer, 37°C, 2000 rpm for 15 min. Samples were then further homogenized using a 20-gauge syringe. Using this homogenized brain tumor tissue, we then performed liquid-liquid extraction using tert-butyl methyl ether (TBME) containing internal standard. The internal standard used for panobinostat was 50 nM panobinostat d8 (Sussex Research catalog SI160030) or for belinostat 250 nM oxamflatin (MedChemExpress catalog HY-102033). Three rounds of TBME liquid-liquid extractions were performed prior to evaporating the TBME buffer via SpeedVac. Finally, samples were resuspended in HPLC-grade methanol prior to being run on Thermo Vanquish UHPLC coupled to a TSQ Plus tandem mass spectrometer, and then quantified against a matrix-matched calibration curve. Matrix-matched calibration samples were prepared by adding standards to TBME extracts from vehicle-treated brain tumor tissue prior to evaporation via SpeedVac. Standards were purchased from MedChemExpress for both belinostat (catalog HY-10225) and panobinostat (catalog HY-10224).

### Immunohistochemical assessment of YAP and TAZ expression in patients with glioma.

Formalin-fixed, paraffin-embedded (FFPE sections from patient-derived IDH^wt^ and IDH^mut^ gliomas were sectioned, stained, and semiquantitatively scored for YAP or TAZ protein expression by a board-certified neuropathologist (CMH), based on the following scale: 0=no staining; 1=weak; 2=moderate; 3=strong; 4=very strong. Instances were scored while blinded to IDH^mut^ status. For immunohistochemical staining, anti-YAP1 (catalog 14074) and anti-TAZ (catalog 72804) antibodies were purchased from Cell Signaling Technology.

### Generation of IDH^mut^ glioma models with constitutively active YAP or TAZ.

Lentiviral transduction was used to generate isogenic in vitro models of constitutively active YAP- or TAZ-expressing IDH^mut^ glioma. Briefly, 0905 and TS603 cultures were transduced with lentiviral particles carrying control lentivirus (RFP/GFP), constitutively active YAP (YAP 5SA), or constitutively active TAZ (TAZ 4SA), along with a blastidicin resistance gene. These lentiviral particles were purchased from VectorBuilder. Forty-eight hours after transduction, cultures were treated with 5 μg/μL blasticidin. After 10 days of blasticidin treatment, nontransduced controls had completely lost viability while transduced cultures retained viability. YAP and TAZ expression were validated via western blot using anti-YAP1 (catalog 14074) and anti-TAZ (catalog 72804) antibodies from Cell Signaling Technology.

### DepMap analysis.

DepMap (https://depmap.org/portal), a database containing multidimensional cell culture data relating to genomics, transcriptomics, and drug sensitivity, was utilized to assess the association between *YAP1/WWTR1* gene expression and HDAC function and/or inhibition (18).

### TCGA analysis.

The UCSC Xena browser (53) was used to compare *HDAC1-11*, *YAP1*, and *WWTR1* gene expression in the TCGA-GBMLGG cohort, with corresponding *YAP1* and *WWTR1* DNA methylation levels included. After removing patients with “null” values, *YAP1* and *WWTR1* gene expression is plotted as median values with upper and lower quartiles denoted. Statistical significance was assessed using a 1-way ANOVA test. For *HDAC1-11*, differences in median Log_2_FCs between IDH^mut^ and IDH^wt^ patient samples were calculated and presented as a waterfall plot.

### Statistics.

Data collection, processing, and statistical analysis were performed with Microsoft Excel and GraphPad Prism V10.1.0 unless otherwise noted. For in vitro dose-response assays, nonlinear regression models were used to determine relative IC_50_ values. Survival between in vitro cohorts was compared via log-rank test. Two-tailed Student’s *t* tests, 2-way ANOVA with post hoc Tukey’s test for multiple comparisons, and Spearman’s correlation analyses were performed as appropriate.

### Study approval.

All animal studies used for this research complied with and were approved by the Institutional Animal Care and Use Committee at Northwestern University under protocol ID IS00002518.

### Data availability.

Raw RNA-Seq for panobinostat-treated glioma cultures are in GEO at GSE308634. Raw RNA-Seq data for belinostat-treated glioma cultures are in GEO at GSE308636. Raw ChIP-Seq data are in GEO at GSE308631. All raw values used in the preparation of this manuscript in the Supporting Data Values file.

## Author contributions

TKS designed and performed experiments, performed data analysis, and wrote the manuscript. MM helped design and conduct experiments, in addition to providing support for data analysis. WW, AS, and KM helped perform experiments and assisted with data analysis. JNS assisted with experimental design and provided critical glioma models. CMH designed experiments, assisted with data analysis, and wrote/revised the manuscript. RC helped perform experiments and assisted with data analysis along with WW, AS, and KM.

## Funding support

This work is the result of NIH funding, in whole or in part, and is subject to the NIH Public Access Policy. Through acceptance of this federal funding, the NIH has been given a right to make the work publicly available in PubMed Central.

- NIH R01NS118039, R01NS117104, and R01NS102669 (CMH).
- NIH P30CA060553 and P50CA221747.
- American Cancer Society fellowship award, PF-23-1156935-01-ET (TKS).

## Supplementary Material

Supplemental data

Supplemental table 1

Supplemental table 2

Supplemental table 3

Supplemental table 4

Supplemental table 5

Supporting data values

## Figures and Tables

**Table 1:**
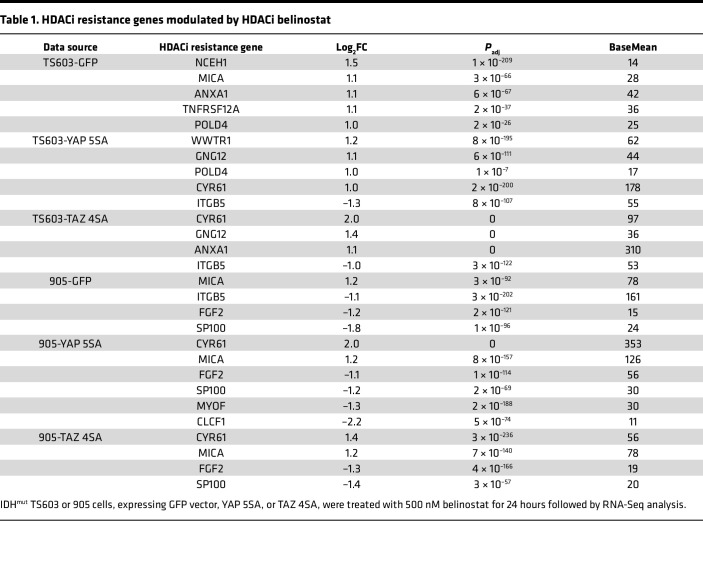
HDACi resistance genes modulated by HDACi belinostat

## Version 1 10/09/2025

In-Press Preview

## Version 2 11/24/2025

Electronic publication
