# Supplementary material for: Methylation-induced suppression of YAP/TAZ confers sensitivity to HDAC inhibitors in high-grade IDH mutant gliomas
Source: JCI Insight. 2025 Oct 9;10(22):e195385. doi: 10.1172/jci.insight.195385 (PMC12643497; doi:10.1172/jci.insight.195385)
Supplement: Supplemental data [file jciinsight-10-195385-s084.pdf]

**Supplementary data for**

# Methylation-induced suppression of YAP/TAZ confers sensitivity to histone deacetylase inhibitors in high grade IDH mutant gliomas

Thomas K. Sears[1], Matthew McCord[2], Wenxia Wang[1], Alicia Steffens[1], Kathleen McCortney[1], Rahul Chaliparambil[1], Jann N. Sarkaria[3], and Craig M. Horbinski[4]

**Affiliations:**

[1]Department of Neurological Surgery, Feinberg School of Medicine, Northwestern University, Chicago IL

[2]Department of Pathology, University of Virginia School of Medicine, Charlottesville VA

[3]Department of Radiation Oncology, Mayo Clinic, Rochester MN

[4]Department of Laboratory Medicine and Pathology, Mayo Clinic Florida, Jacksonville FL

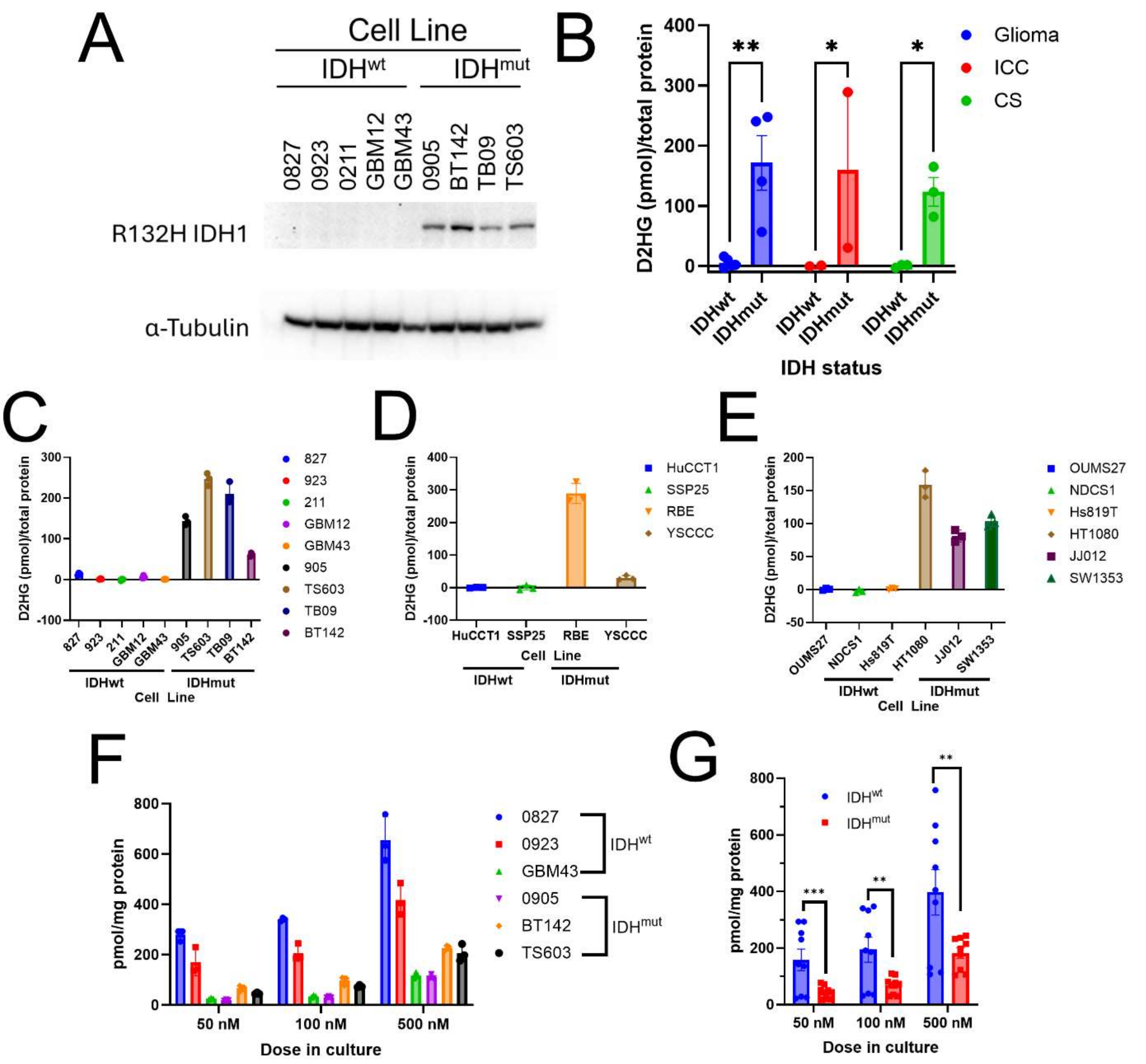


**Supplementary Figure 1: Characterization of IDH status and drug uptake in our cell culture models of IDH$^{wt}$ and IDH$^{mut}$ glioma.** (**A**) Western blot validation of R132H IDH1 mutations in IDH$^{mut}$ glioma cells. (**B-E**) D2HG quantification via an enzymatic assay in IDH$^{wt}$ and IDH$^{mut}$ glioma (**C**), intrahepatic cholangiocarcinoma (**D**), and chondrosarcoma (**E**) cultures consolidated in **B**. 2-way ANOVA with Bonferonni correction for multiple comparisons. **P* < 0.05; ***P* < 0.01. Bars represent mean +/- SEM (n=3 biological replicates). (**F**) Quantification of intracellular panobinostat concentrations via tandem mass spectrometry for the evaluation of drug uptake in IDH$^{wt}$ and IDH$^{mut}$ glioma cultures. Cultures were treated with 50, 100, or 500 nM panobinostat for 5 hours. Bars represent mean +/- SEM (n=3 biological replicates). (**G**) Consolidation of quantification data from (**F**) by IDH status for each dose of panobinostat. Multiple unpaired t-tests with Benjamini-Hochberg correction for multiple comparisons. ***P* < 0.01; ****P* < 0.001. Bars represent mean +/- SEM (n=3 biological replicates).

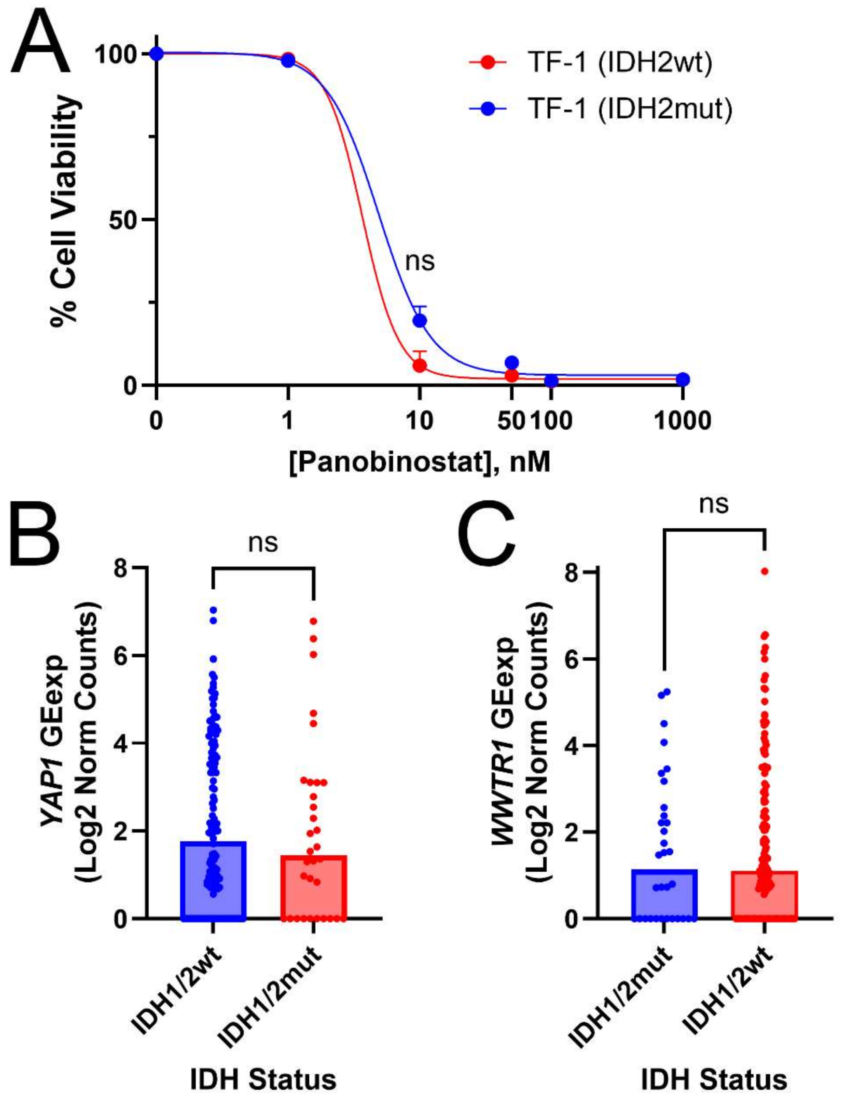


**Supplementary Figure 2: HDACi activity against IDH$^{wt}$ and IDH$^{mut}$ AML cells.** (A) Dose-response curve for IDH2$^{wt}$ and IDH2$^{mut}$ TF-1 AML cells in response to 24 hrs panobinostat. (B) TCGA data for *YAP1* mRNA by IDH$^{mut}$ status in AML. (C) TCGA data for *WWTR1*/TAZ mRNA by IDH$^{mut}$ status in AML.

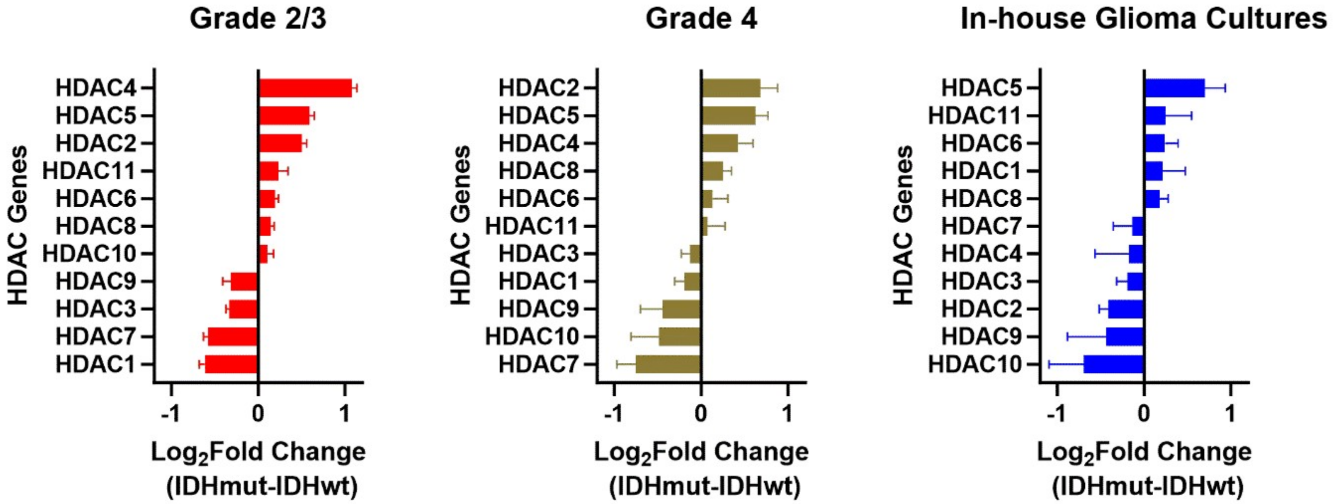


**Supplementary Figure 3: *HDAC* gene expression in IDH$^{mut}$ gliomas.** Gene expression for *HDAC1-11* using TCGA data for Grade 2/3 gliomas (TCGA-LGG) or Grade 4 gliomas (TCGA-GBM) in relation to our in-house RNA-Seq data. Data is plotted as the difference in mean *HDAC* gene expression between IDH$^{mut}$ and IDH$^{wt}$ gliomas for each dataset.

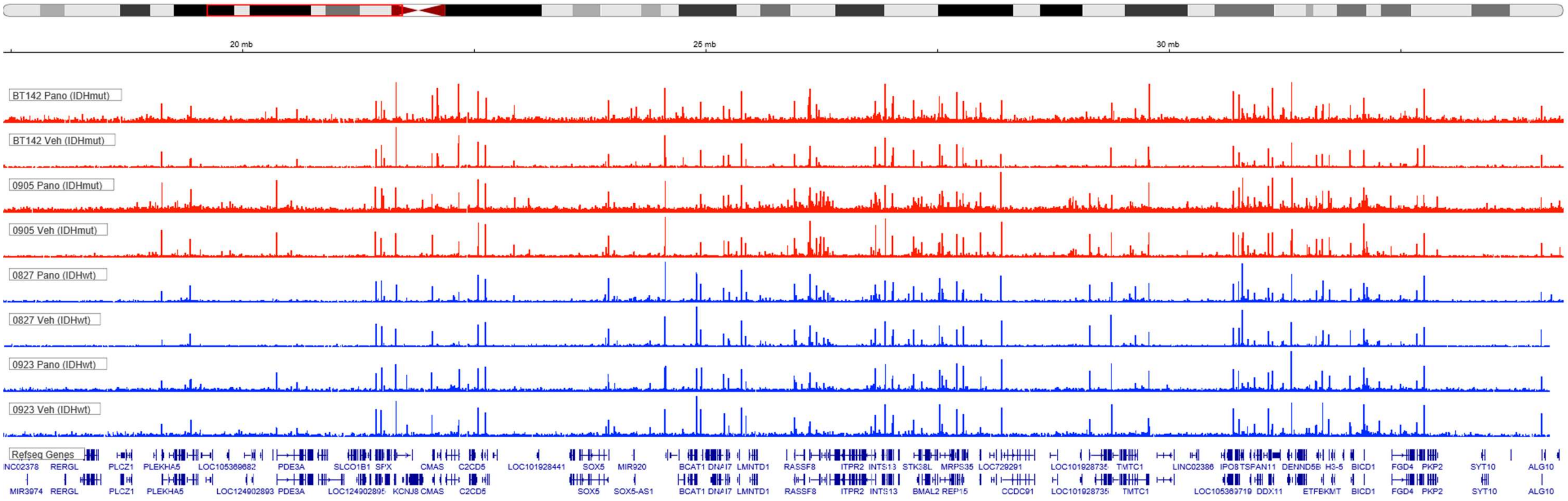


**Supplementary Figure 4: H3KAc ChIP tracks from a 20mb region of chromosome 12.** This shows widespread, low-level increases in histone acetylation primarily in $IDH^{mut}$ (red) but not $IDH^{wt}$ (blue), glioma cells.

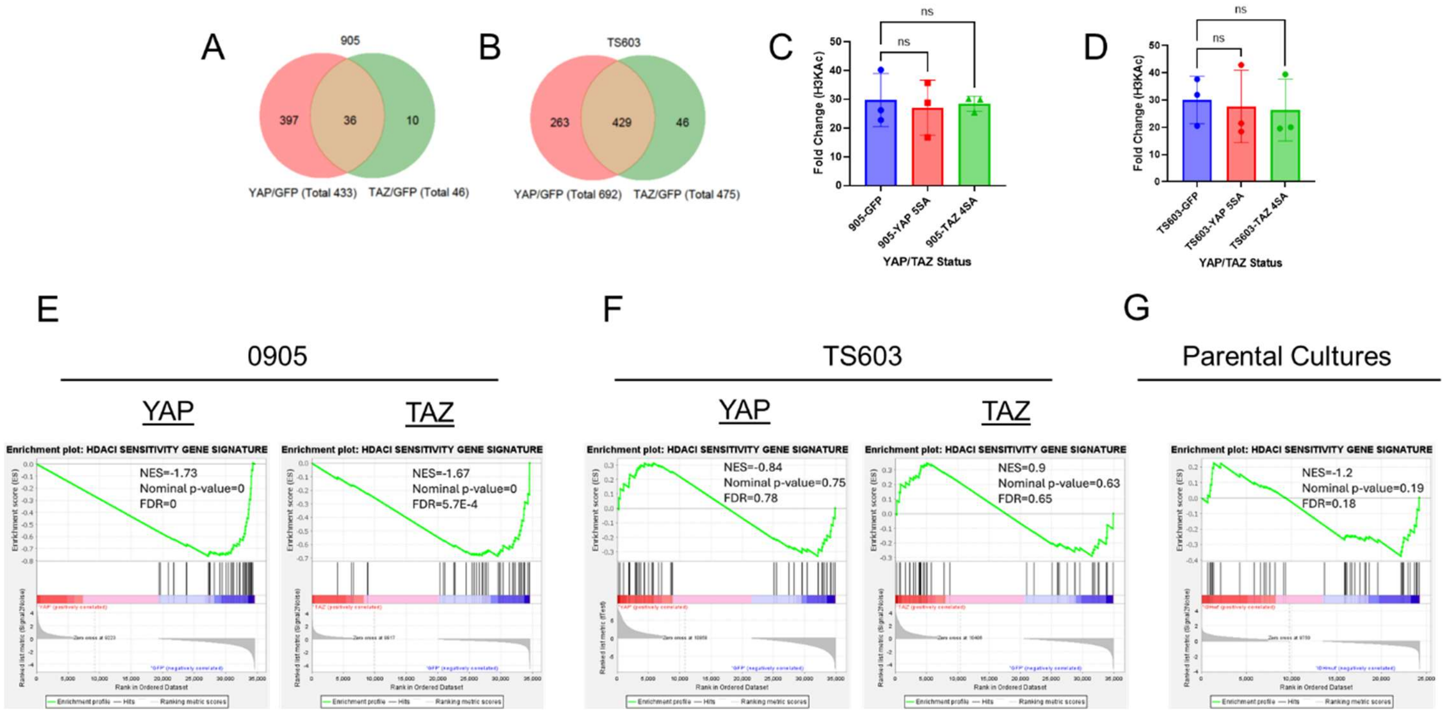


**Supplementary Figure 5: Associated RNA-Seq and H3KAc data from YAP- and TAZ-overexpressing $IDH^{mut}$ glioma cultures.** (**A-B**) RNA-Seq was performed on our TAZ 4SA- and YAP 5SA-overexpressing 0905 (**A**) and TS603 (**B**) cultures. Venn diagrams show significant differentially expressed genes with a $Log_2FC > 1$, and how many of these gene overlap between our TAZ- and YAP-overexpressing 0905 and TS603 cultures. (**C-D**) H3KAc ELISA of YAP- or TAZ-expressing 0905 (**C**) and TS603 (**D**) $IDH_{mut}$ glioma cultures treated with 10 nM panobinostat for 24 hours. One-way ANOVA with Bonferonni correction for multiple comparisons. Bars represent mean +/- SEM (n=3 biological replicates). (**E-G**) GSEA of a HDACi Sensitivity Gene Signature in YAP- and TAZ-overexpressing 0905 (**E**), TS603 (**F**), and parental cultures (**G**).

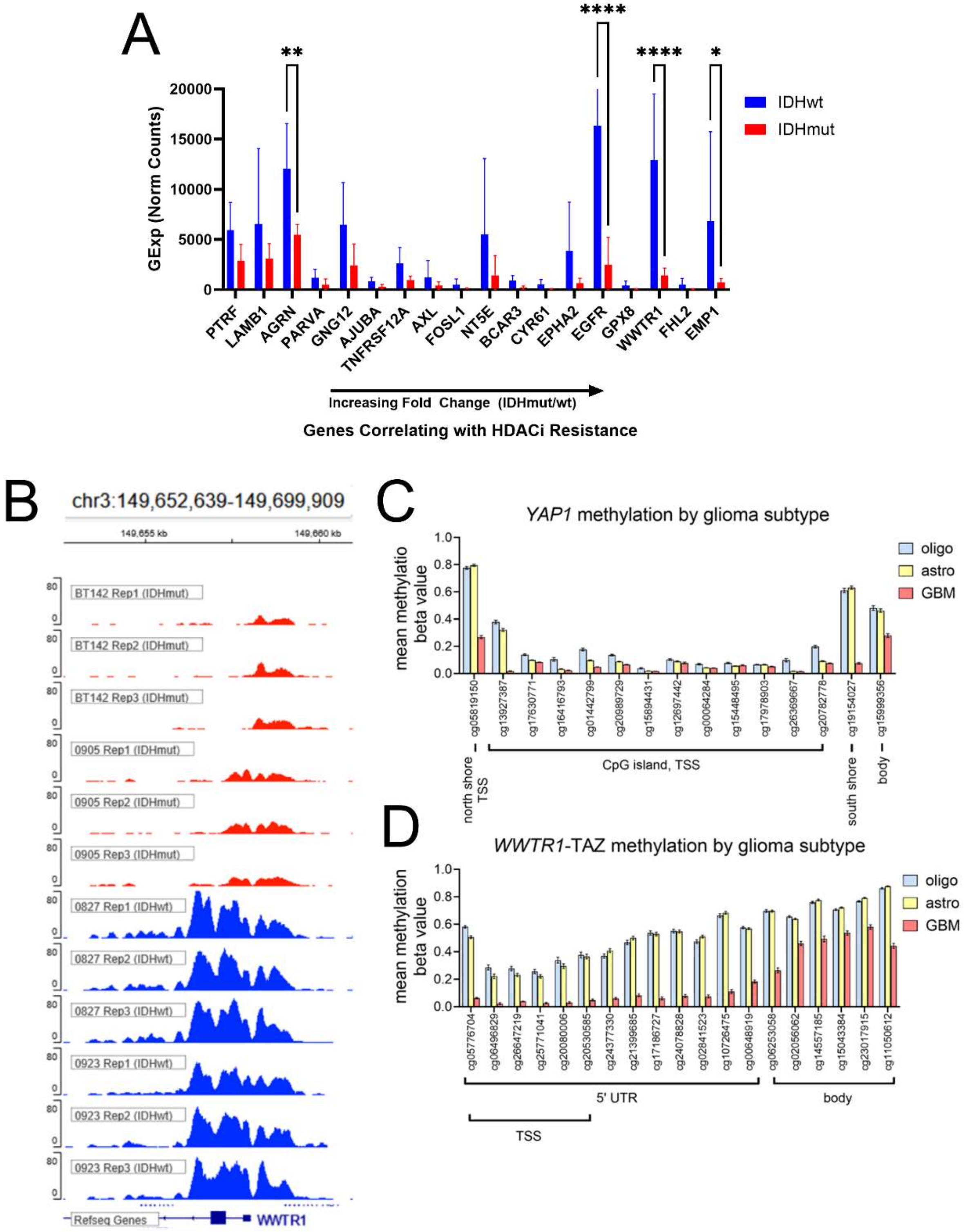


**Supplementary Figure 6: WWTR1 and TAZ are downregulated in IDH$^{mut}$ glioma.** (**A**) Gene expression (via RNA-Seq) of the top 20 genes associated with panobinostat resistance as determined by DepMap. Multiple unpaired t-tests with Benjamini-Hochberg correction for multiple comparisons. *$P$ < 0.05; **$P$ < 0.01; ****$P$ < 0.0001. Bars represent mean +/- SEM (n=3 biological replicates). (**B**) H3KAc ChIP tracks showing the *WWTR1* gene promoter in IDH$^{wt}$ and IDH$^{mut}$ glioma cultures. (**C**, **D**) TCGA-GBMLGG data of *WWTR1* (**C**) and *YAP1* (**D**) methylation based on molecular subtype.

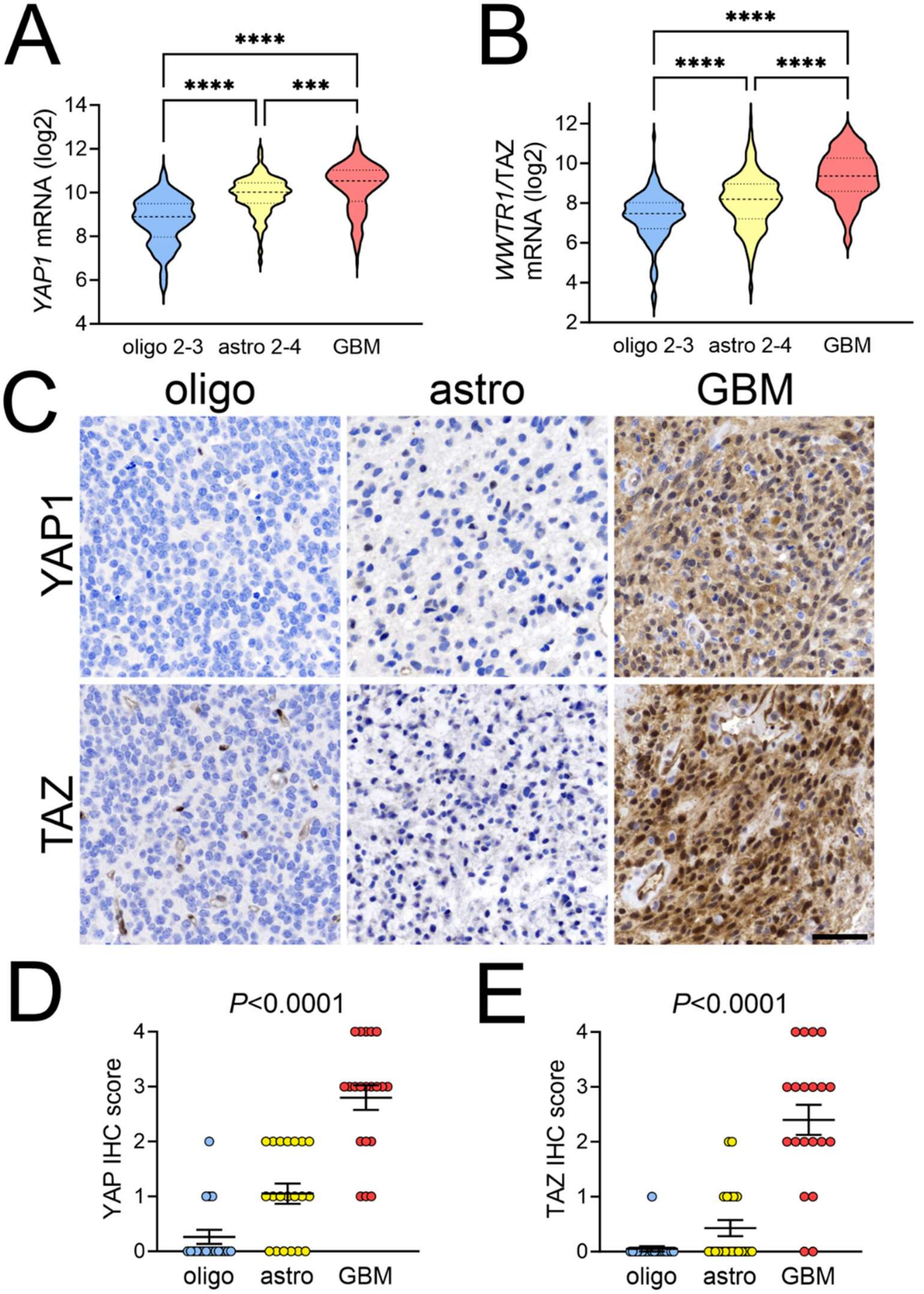


**Supplementary Figure 7: Immunohistochemical analysis of YAP1 and TAZ protein levels in patient glioma samples with IDH$^{mut}$ oligodendroglioma, IDH$^{mut}$ astrocytoma, or IDH$^{wt}$ GBM.** (**A**) *YAP1* and (**B**) *WWTR1*-TAZ mRNA levels in TCGA IDH$^{mut}$ grade 2-3 oligodendrogliomas, IDH$^{mut}$ grade 2-4 astrocytomas, and IDH$^{wt}$ GBM. ****P*<0.001, *****P*<0.0001 by one-way ANOVA with post hoc Tukey's tests. (**C**) Representative photomicrographs of YAP and TAZ IHC in IDH$^{mut}$ oligodendroglioma and astrocytoma vs. IDH$^{wt}$ GBM. Scale bar=50 microns. Semiquantitative scoring of (**D**) YAP and (E) TAZ IHC in 20 IDH$^{mut}$ oligos, 21 IDH$^{mut}$ astros, and 20 IDH$^{wt}$ GBM while blinded to genotype. *P*<0.0001 by Kruskal-Wallis test.

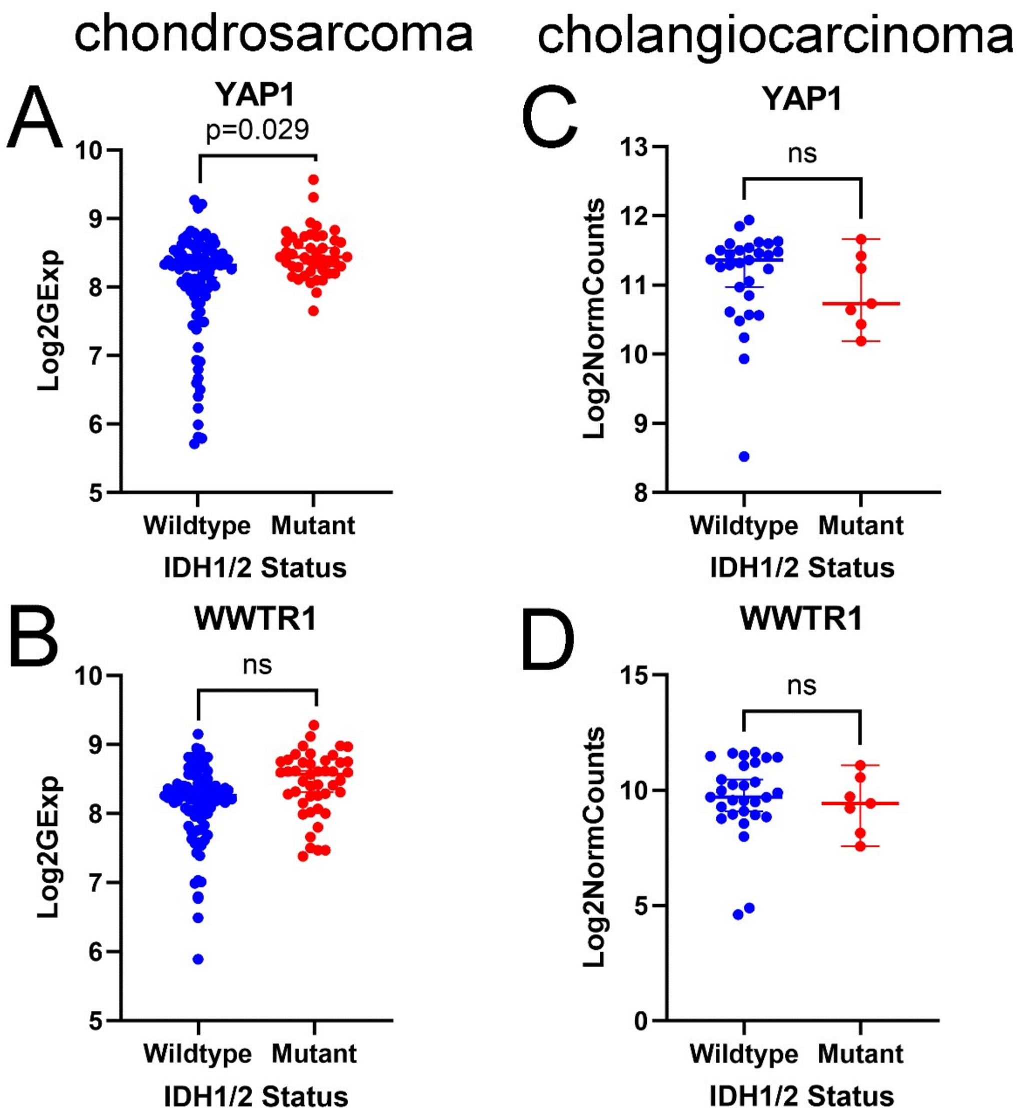


**Supplementary Figure 8: *YAP1* and *WWTR1* mRNA in chondrosarcoma and cholangiocarcinoma.** (**A-B**) Microarray data from IDH$^{wt}$ (n=99) and IDH$^{mut}$ (n=45) chondrosarcoma patient samples showing gene expression of *YAP1* (**A**) and *WWTR1* (**B**). Multiple unpaired t-tests with Benjamini-Hochberg correction for multiple comparisons. (**C-D**) TCGA-CHOL RNA-Seq data from IDH$^{wt}$ (n=29) and IDH$^{mut}$ (n=8) cholangiocarcinoma patient samples showing gene expression of *YAP1* (**C**) and *WWTR1* (**D**). Multiple unpaired t-tests with Benjamini-Hochberg correction for multiple comparisons.

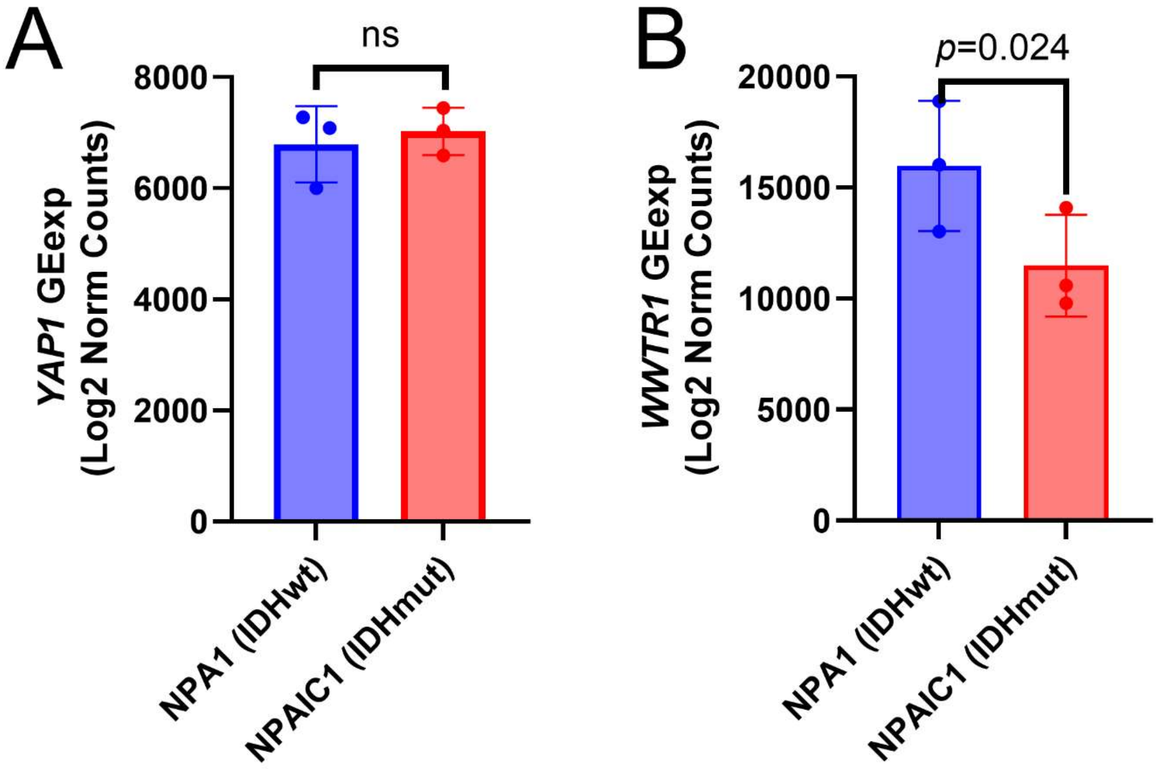


**Supplementary Figure S9: YAP and TAZ expression in mouse models of IDH$^{wt}$ vs. IDH$^{mut}$ glioma.** (A) *YAP1* mRNA in IDH$^{wt}$ NPA1 vs. IDH$^{mut}$ NPAIC1 glioma. (B) *WWTR1* mRNA in IDH$^{wt}$ NPA1 vs. IDH$^{mut}$ NPAIC1 glioma. *P* calculated via student's t-test.
